# Three-in-one: exploration of co-encapsulation of cabazitaxel, bicalutamide and chlorin e6 in new mixed cyclodextrin-crosslinked polymers†

**DOI:** 10.1039/d3ra01782f

**Published:** 2023-04-06

**Authors:** Elisabetta Pancani, Daniele Veclani, Marco Agnes, Arianna Mazza, Alessandro Venturini, Milo Malanga, Ilse Manet

**Affiliations:** a Institute for Organic Synthesis and Photoreactivity (ISOF), National Research Council of Italy (CNR) Via P. Gobetti 101 I-40129 Bologna Italy ilse.manet@isof.cnr.it; b CycloLab, Cyclodextrin R&D Ltd. Budapest Hungary

## Abstract

We explored a series of cyclodextrin (CyD) polymers composed either of a single CyD type or a mixture of two CyD types to encapsulate simultaneously different compounds with potential therapeutic interest for multimodal prostate cancer treatment. New mixed CyD polymers were prepared in alkaline water starting from the naturally occurring monomers and a low-cost crosslinking agent. Batches of 200 g of polymer were easily obtained. By means of optical spectroscopy we proved the co-encapsulation of 3 compounds in the polymers: the drugs cabazitaxel (CBX) and bicalutamide (BIC), and the photosensitizer chlorin e6 (Ce6). pβCyD and mixed pαβCyD polymers performed best for single drug solubilization. In the co-encapsulation of BIC and CBX by pβCyD and pαβCyD, pβCyD stands out in drug solubilization ability. Avoiding the use of organic solvents, it was possible to dissolve up to 0.1 mM CBX with 10 mg ml^−1^ pβCyD polymer and, with 100 mg ml^−1^, even 1.7 mM BIC, a 100-fold improvement compared to water. Spectroscopic studies afforded the binding constants of CBX and BIC with pβCyD forming complexes of 1 : 2 stoichiometry (drug : CyD) and CBX displayed significantly higher affinity. Also DFT calculations suggested that the drugs are more stable when complexed by two CyD units. Ce6 could be encapsulated simultaneously with the other two drugs in pβCyD and, most importantly, is able to produce singlet oxygen efficiently. Thanks to a single inexpensive CyD-based polymer we were able to produce a three-in-one platform for future implementation of combined chemotherapy and photodynamic therapy. These achievements are most relevant as nanomedicines are continuously proposed but their potential for translation to the pharma industry is compromised by their limited potential for industrial upscale.

## Abstract 2

Three-in-one: a single bCyD polymer easily prepared in water is used to co-encapsulate cabazitaxel and bicalutamide with chlorin e6 affording a nanoplatform to implement multimodal cancer therapy.

## Graphical abstract

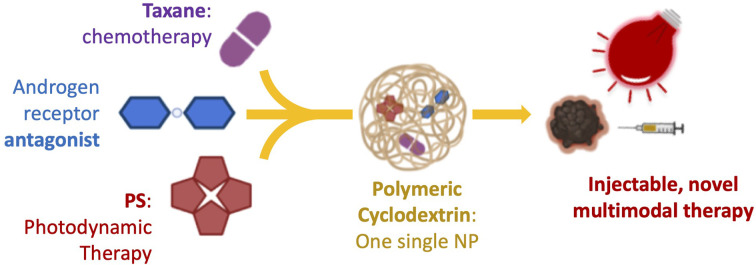

## Introduction

In the male population Prostate Cancer (PC) remains a burden accounting for 15% of all new cancer cases yearly.^1^ Localized PC can be successfully cured by radiation or surgery while locally advanced and metastatic PC diseases are treated mainly with androgen deprivation therapy (ADT).^2–4^ ADT aims at blocking the biological processes involving the androgen receptor (AR) protein by reducing the amount of androgen ligands like testosterone to so-called “castration levels” (*i.e.* very low levels) or by administrating antagonists of androgens. ADT generally induces significant tumor regression, however, hormone-sensitive PC often evolves into a castration-resistant (CR) state, with few therapeutic alternatives currently available.^5,6^ So there is an urgent need for the development of new treatment options that will likely require personalized combination therapy with hormonal agents, chemotherapeutics and new drugs targeting metabolic pathways specifically activated in PC. Administration of multiple therapeutic agents remains challenging, however, in this context nanotechnology applied to drug delivery can offer a series of advantages such as the possibility to combine in a unique carrier system more than one therapeutic agent and to increase the bioavailability of the single components.^7–10^ Furthermore, it has been reported that several nanocarriers can afford implementation of classical chemotherapy with other types of treatment like photodynamic therapy (PDT).^11–15^ Combination therapy aims at the synergic action of different therapeutics targeting more than one metabolic pathway in order to maximize the impact of the treatment and eventually improve the therapeutic outcome. Up to now, a low number of drug nanocarrier systems implementing combination therapy have reached the stage of clinical trials and only few of them have been approved by the regulatory agencies. Some of the aspects that have been so far neglected by the scientific community at the early design stage of drug nanocarriers are (i) the consequences of small chemical changes in the nanoparticle (NP) composition that affect drastically pharmacokinetic and -dynamic profile, (ii) the potential for production scale-up at affordable costs selecting, for example, inexpensive and/or biocompatible reagents, (iii) selection of preparation protocols that can assure reproducibility of different production batches as well as sustainability thanks to circular economy production processes.^16–20^

**Scheme 1:**
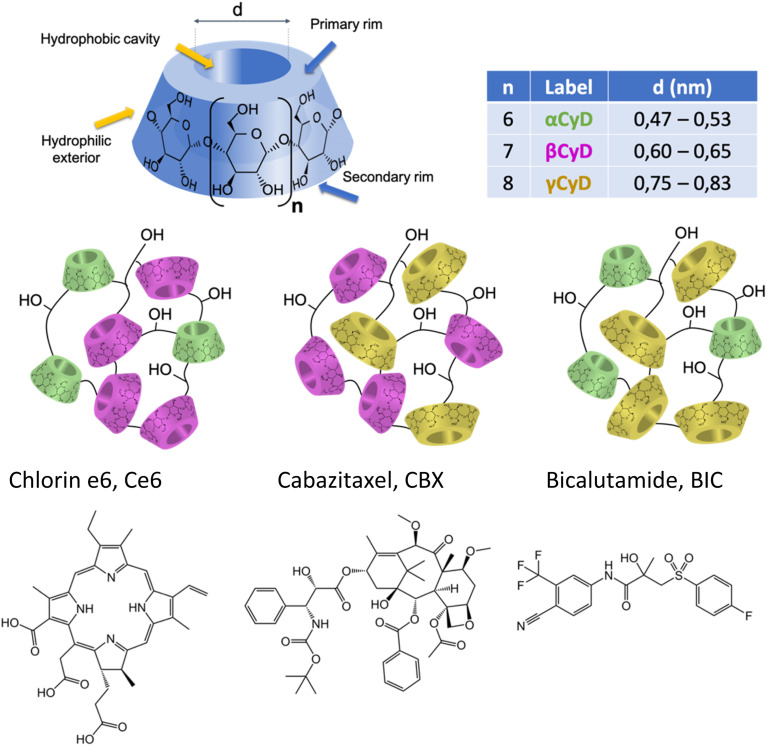
CyD structure and chemical structure of the target molecules for multimodal therapy.

Aware of this context we recently addressed the challenge of the implementation of multimodal therapy for CRPC focusing on polymers of cyclodextrins (CyDs) organizing in nanoparticles (NP) as valuable tool to opportunely assemble the various molecular players. CyDs are water soluble, biocompatible cyclic oligosaccharides, made of α-d-glucopyranose units joined by α(1–4) linkages (Scheme 1).^21^ Among the large variety of CyD-based materials, CyD polymers were selected for several reasons. First, CyD polymers can be prepared following a polycondensation procedure with a crosslinking agent like epichlorohydrin (EPI) in water which is a very appealing feature for sustainable development of new drug carriers or therapeutics.^22,23^ Moreover, the manufacture requires a very low number of reagents with accessible costs keeping the synthesis cheap and the natural CyD starting materials are enzymatically produced.^24–26^ The preparation of these polymeric compounds is a standardized process at laboratory-scale and batch-to-batch reproducibility as well as the in-depth characterization of these derivatives has been largely achieved. Some EPI crosslinked CyD polymers are nowadays commercially available as fine chemicals produced on 100 g scale. Secondly, they are able to associate drugs much more efficiently than the monomeric CyDs, known excipients, and to incorporate two or more therapeutics simultaneously assembling in NPs.^26–28^ The CyD polymer NPs can help overcome standard drug shortcomings such as low solubility and poor stability in pharmaceutical vehicles for drug administration.^29,30^ Last but not least, they have also far superior solubility in water; βCyD can reach a maximum concentration of 0.01 M in water while the epichlorohydrin-crosslinked βCyD polymer (pβCyD) can bring the βCyD content up to 0.1 M.^31,32^ Overall, these features make CyD polymers very attractive for the development of new drug delivery systems. Our first experience in the use of pβCyD to combine therapeutic agents consisted in the co-encapsulation of a photosensitizer (PS) Zn(ii) phthalocyanine, with a nitroaniline NO-donor covalently linked to a benzofurazan fluorophore.^33^ Assembled in the polymer as NPs both components acting independently were able to produce singlet oxygen (^1^O_2_) and the NO radical upon Near Infrared and blue light irradiation, respectively, eventually provoking melanoma cell death. The system was traced *in vitro* by means of the PS and NO-donor label and localized mainly in the cytoplasm. More recently, we reported the use of pβCyD to assemble the antibiotic ethionamide together with a synthetic booster in a unique NP system opening new avenues for Microsprayer® administration of the drug combination in tuberculosis treatment. The system administered with the Microsprayer® resulted to be effective in mice with drug and booster acting in synergy and reducing with several log units the bacterial load.^31,34^

In this study our goal was to explore the capacity of various CyD polymers for the co-encapsulation of three components in view of the implementation of chemotherapy and PDT. We explored the use of classical CyD polymers made from one type of CyD (α, β and γCyDs) as well as new mixed polymers combining two different types (α/β, α/γ, and β/γ). Indeed, we expect the loading capacity for different therapeutic agents with diverse structural characteristics to depend on the CyD cavity size (Scheme 1). Up to now the mixed CyD polymers have not been explored as delivery systems for multiple therapeutic agents.

We focused on two drugs, structurally very different, proposed for the treatment of CRPC, cabazitaxel (CBX), a tubulin-blocking taxane and bicalutamide (BIC), an androgen receptor (AR) antagonist (Scheme 1). These drugs display serious drawbacks due to their low aqueous solubility and the onset of severe side effects, forcing administration in low doses to guarantee tolerability. Our choice is rationalized by some recent publications on the combination of cabazitaxel and AR antagonist as a tool to resensitize taxane-resistant PC to the drug.^35,36^ The solubilizing power of different CyD polymers for the two drugs alone and in combination has been assessed. Stability of the drug loaded polymers has been explored. For the best performing polymers, the drug binding constants in aqueous solution were determined. The particle size of the loaded polymers organizing in NPs and the NP stability and possible aggregation were assessed. The study was completed encapsulating also the PS chlorin e6 (Ce6, Scheme 1) within the carrier with the two drugs and assessing its ability to produce singlet oxygen, ^1^O_2_. In order to obtain more information on the host–guest complexation process, computational chemistry calculations on the inclusion of CBX and the R and S isomer of BIC in the βCyD monomer model with a 1 : 1 and 1 : 2 (guest : CyD) stoichiometry, were carried out using DFT methods.^37^

## Experimental methods

### Materials

Cabazitaxel (CBX, purity 99.92%, CAS 183133-96-2) and bicalutamide, (BIC, racemic mixture, purity 99.85%, CAS 90357-06-5) were purchased from MedChemExpress; chlorin e6 sc-263067 (purity > 95%, Santa Cruz Biotechnology, Inc.) was purchased from Santa Cruz Biotechnology, Inc. The polymers were produced by crosslinking CyD monomers under strongly alkaline conditions in the presence of epichlorohydrin (EPI). The pβCyD NPs, were recovered by ultrafiltration followed by freeze-drying. ^1^H NMR spectroscopy was used to determine the CyD content. The polymers contain 60–70% w/w of CyD, have a molecular weight of 50–60 kDa determined by means of size exclusion chromatography. DLS with a Malvern Nanosizer system was used to determine the particle size of the loaded polymers and to check on possible aggregation over time. All solvents were of analytic or spectroscopic grade.

### Spectroscopic study of the binding equilibria

A series of solutions with low fixed drug concentration and different amounts of polymer in aqueous solutions ranging from and 0.5–2 mg ml^−1^, respectively, were prepared. CBX and BIC were first solubilized in ethanol. Then, the different aqueous polymer solutions were added to dried films of known volumes CBX and BIC and kept stirring for 1 night at room temperature under light protection. Eventually the mixtures were studied by means of optical spectroscopy. In particular, absorption, fluorescence and circular dichroism (CD) were used.

The best complexation model and the association constants were determined by means of global analysis of multiwavelength titration data from a set of spectra corresponding to different host–guest mixtures, using the program ReactLabTM Equilibria (Jplus Consulting Pty Ltd). The procedure is based on singular value decomposition (SVD) and non-linear regression modelling by the Levenberg–Marquardt method. The analysis also affords the individual absorption, CD and/or fluorescence spectra of the associated species. In order to fit the data different binding models were explored with either one complex of 1 : 1 or 1 : 2 stoichiometry or two complexes with 1 : 1 and 1 : 2 stoichiometry (drug : CyD); as polymers contain more than 25 CyD units, the CyD concentration was used and not the polymer amount. This is an approximation that can afford affinity constants for the CyD unit, but ignores other binding sites possibly present in the polymer. The equilibrium constants listed in Table 2 are the following, with *G* the symbol used for the guest. The equilibrium constant of the 1 : 1 stoichiometry complex, *K*_1:1_ (M^−1^):
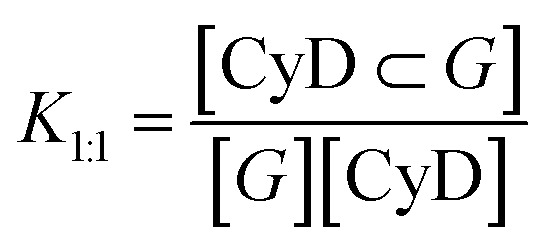


The overall equilibrium constant of the complex with 1 : 2 stoichiometry, *K*_1:2_ (M^−2^)
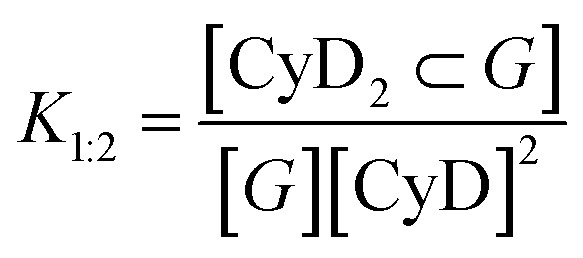


### Optical spectroscopic measurements

Absorption spectra were recorded on a PerkinElmer Lambda 950 spectrophotometer. 1 cm cuvettes were used and solvents were used as reference. Circular Dichroism (CD) spectra were obtained with a Jasco J-715 spectropolarimeter. The spectra of the mixtures were registered using solvent or polymer solutions as reference. To reduce the signal to noise ratio 4 spectra were accumulated at 50/100 nm min^−1^ velocity. Fluorescence spectra of BIC and Ce6 were registered on a Edinburgh FLSP920 spectrofluorimeter with excitation in isosbestic point at 270 or 600 nm, respectively, registering spectra with 1 nm steps and 0.5 or 1 s dwell time. Slits were kept narrow to 2–3 or 4–6 nm bandwidth in excitation and emission, respectively. Where necessary, a cut-off filter was used. Right angle detection was used. All the fluorescence measurements were carried out at room temperature in quartz cuvettes with path length of 1 cm. Steady state fluorescence spectra were registered in air-equilibrated solutions. A N_2_ cooled Hamamatsu photomultiplier was used to measure the steady-state emission of ^1^O_2_ in the 1150–1450 nm range accumulating 4 scans to improve S/N ratio.

Fluorescence lifetimes of BIC were measured in air-equilibrated solutions by means of a time-correlated single photon counting system with a resolution of 55 ps per channel (IBH Consultants Ltd, Glasgow, UK). A nanosecond LED source of 273 nm was used for excitation and the emission was collected at right angle at 340 nm (bandwidth 4 nm). A cut-off filter 305 was used. An opaque glass plate is used to register the Instrument Response Function collecting photons at 300 nm using the 273 nm LED light source. 4000 counts were collected in the maximum intensity channel (channel width of 55 ps) corresponding to a total photon count ranging of *ca.* 95 000 cts per decay. Fluorescence decays of Ce6 were measured in air-equilibrated solutions for excitation at 637 nm (Hamamatsu pulsed laser with 1 MHz repetition rate). Photons were detected in right angle geometry at 670 nm with a cut-off filter at 645 nm. 2000 counts were collected in the maximum intensity channel corresponding to a total photon count ranging of 150 000–200 000 cts per decay.

Fluorescence decay profiles were analyzed with a least-squares method, using multiexponential decay functions (eqn (1)) and deconvolution of the instrumental response function. Upon deconvolution form the instrumental response function the lifetime lower limit is 200 ps. The software package for the analysis of the emission decays was provided by IBH Consultants Ltd Fluorescence intensity decay profiles were fitted using a multi-exponential function and deconvolution of the instrumental response function. Fitting yields both lifetime *τ*_*j*_ and pre-exponential factor *a*_*j*_ of each emitting species.1
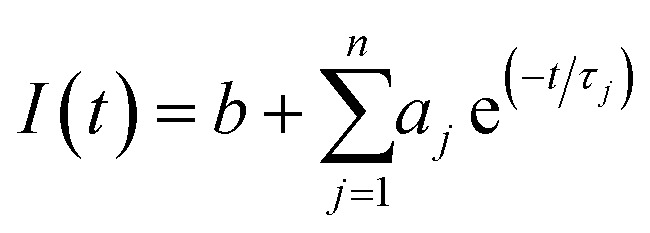


The relative amplitude *α*_*j*_ (eqn (2))and the amplitude weighted average fluorescence lifetime, 〈*τ*〉 (eqn (3)) are calculated according to the following equations:2
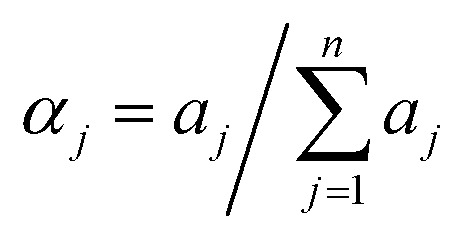
3
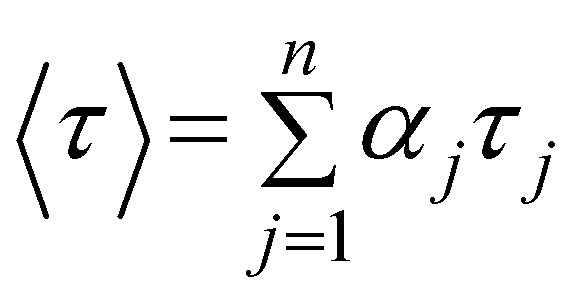


If the multi-exponential decay is due to only one fluorophore such as Ce6, the relative amplitude *α*_*j*_ equals the fractional concentration of each fluorophore species. For the fluorescence titrations a global analysis was performed of all decays including that of the fluorophore alone. The DAS software package provided by IBH Consultants Ltd was used to build a file with all decays. Next one decay was fitted with a 2- or 3-exponential decay function and the parameters of this fit were then optimized for all decays applying the same function. Eventually the converging global analysis afforded the optimized lifetimes together with the preexponential factors *a*_*j*_ for each decay. The lifetime of the free species is introduced as constant and is not further optimized.

### Calculation procedures

All calculations were performed using the ORCA 5.0.3 software package.^38^ Geometry optimization in vacuum were carried out using the PBE GGA functional^39^ with Def2-SVP basis set for all atoms.^40,41^ Dispersion correction has been included employing the Grimme's pair-wise additive method, DFT-D3;^42^ previous works showed that the DFT-D3 method provides reliable results where the non-covalent interactions play an important role.^43–45^ Due to the key role of solvation in influencing thermodynamic parameters, environmental effects (water solvent) have been introduced using a conductor-like polarizable continuum model on optimized geometries obtained in gas phase.^46^ The UV and ECD spectra has been calculated using the TD-DFT method, the same continuum model for the solvent and the wB97X-D3BJ functional.^47^

The interaction energy (IE) for a two-body system (1 : 1 stoichiometry, see Fig. S5†), is defined in eqn (4)

4 IE = *E*_complex_ − (*E*_βCyD_ + *E*_Guest_) + BSSE

For 1 : 2 stoichiometry complexes (Fig. S6†) the equation for IE is defined by eqn (5).

5 IE = *E*_complex_ − (*E*_2βCyD_ + *E*_Guest_) + BSSE

In eqn (4) and (5)*E*_complex_ is the total energy for the clusters, *E*_βCyD_ is the energy of the isolated βCyD host at the coordinated of the cluster, and *E*_Guest_ the energy of the drug at the coordinated of the cluster.

*E*_2βCyD_ is the energy of the two βCyD hosts interacting with each other. All IEs have been corrected by adding the basis set superposition error (BSSE) using the counterpoise method by Boys and Bernardi.^48^ To quantitatively describe the structural changes of the guest and the host molecules, deformation energies (Def) are calculated and defined by eqn (6) and (7) for 1 : 1 and 1 : 2 complexes, respectively. The parameter DEF represents the energy cost to bring each partner from its optimized structure as an isolated species to that in the complex.

6 Def = (*E*_βCyD_ + *E*_Guest_) − (*E*_βCyD_relax_ + *E*_Guest_relax_)

7 Def = (*E*_2βCyD_ + *E*_Guest_) − (*E*_2βCyD_relax_ + *E*_Guest_relax_)

where *E*_βCyD_relax_ is the energy of the relaxed βCyD, *E*_Guest_relax_ is the energy of relaxed drugs.

The analysis of the non-covalent interaction created between host and guest was performed by the independent gradient model (IGM) method, a natural evolution of traditional noncovalent interaction descriptors (NCI).^49,50^ A new electron density descriptor, δ*g*, was computed by IGMPlot as the difference between a non-interacting model (IGM), represented by a virtual upper limit of the electron density gradient (|∇*ρ*^IGM^|), and the interacting system, represented by the true electron density gradient (|∇*ρ*|). IGM, through the definition of molecular units, is able to uncouple the molecular interactions within the units (δ*g*^intra^) from the molecular interactions between the fragments (δ*g*^inter^). The δ*g* descriptor is related to the interaction intensity concept, in 2D δ*g*^inter^ plots (Fig. S8 and S11†) interactions can be classified into categories based on the height of the δ*g*^inter^ peak as follow:

- weak non-covalent interactions: δ*g* peak heights lower than 0.1 a.u.;

- van der Waals (vdW) interactions: δ*g* peak heights 0.02–0.03 a.u.;

- hydrogen bonds (HBs) interactions: δ*g* peak heights: 0.04–0.1 a.u. (HB in water ≈0.06 a.u.);

- pure covalent bond: δ*g* peak heights 0.2–2.5 a.u;

- metal coordination: δ*g* peak heights 0.1 and 0.6 a.u.

**Fig. 1:**
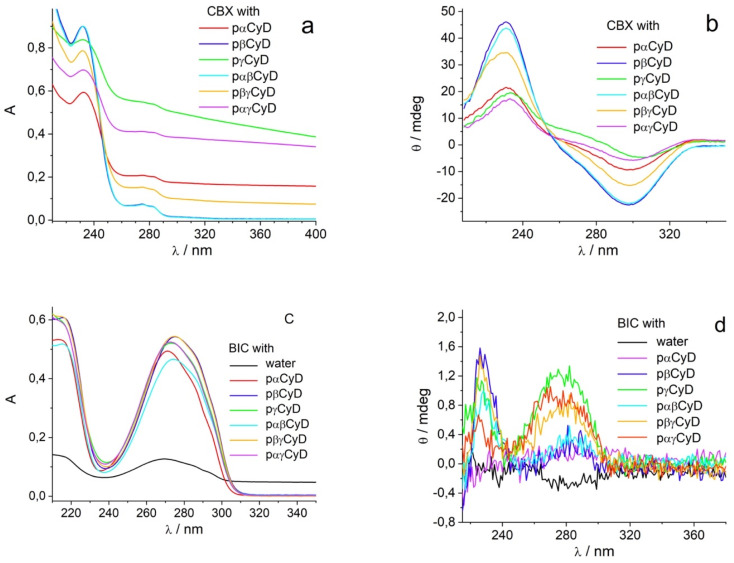
Absorption (a)–(c) and CD (b)–(d) spectra of the solutions of BIC and CBX in the presence of 10 mg ml^−1^ of pCyD; solid amount of 0.05 mg ml^−1^ CBX (60 μM) in 1 cm cuvet (a) and (b); 0.026 mg ml^−1^ BIC (60 μM) in 0.5 cm cuvet (c) and (d).

**Fig. 2:**
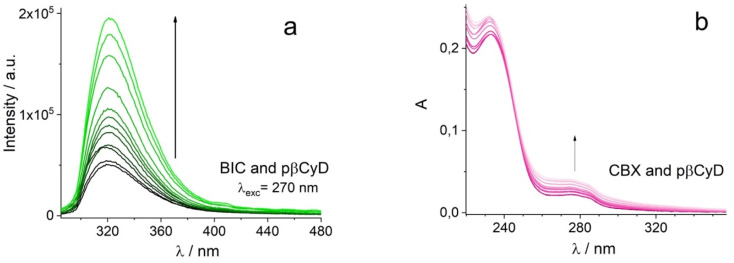
Titration spectra used to define the binding model and calculate the binding constants of the drugs with selected polymers; [BIC] = 4 μM; [CBX] = 8 μM; for the polymer pβCyD: 4–600 μM [βCyD]; (a) and (b) fluorescence spectra of BIC and absorption spectra of CBX.

The sign of the second eigenvalue of the electron density gradient hessian matrix (sign(*λ*_2_)*ρ*) differentiates attractive (*λ*_2_ < 0) interactions from the non-bonding (*λ*_2_ > 0) situations. The next logical step is to use the data from the 2D plot of *g*^inter^(*ρ*) to build 3D plots depicting isosurfaces that represent intermolecular interactions (Fig. 1, 2, S3 and S8†). This δ*g*^inter^(*ρ*) surface can be colored according to the standard NCI approach^49,50^ where green surface shows weak non-bonding or attractive interactions, blue for HBs interactions and or red for nonbonding situations.

## Results and discussion

### Synthesis of the CyD polymers

The polymers were produced by crosslinking the natural CyDs under strongly alkaline conditions with the crosslinking agent EPI (ESI, Scheme 1).^23^ In the case of single α-, β-, γCyD polymer, 250 g CyD (0.26 mol αCyD, 0.22 mol βCyD, 0.19 mol γCyD) was solubilized in alkaline aqueous conditions (160 g NaOH (4.06 mol) in 1.l water). The solution was heated at 70 °C and EPI (145 ml, 1.83 mol) was added dropwise keeping constant the temperature during 2 h. The reaction mixture was cooled down, neutralized with HCl and extensively dialyzed for 3 days (cut-off cellulose membrane 14 kDa). The dialysate was freeze dried (recovered solid ∼ 240 g).

In the case of mixed α/β, α/γ and β/γ polymers the procedure is analogue to the one utilized for the single CyD polymers, but CyDs are blended in equimolar ratio: αCyD (0.1 mol, 97.5 g) and βCyD (0.1 mol, 113.5 g); αCyD (0.1 mol, 97.5 g) and γCyD (0.1 mol, 130 g); βCyD (0.1 mol, 113.5 g) and γCyD (0.1 mol, 130 g). The amount of the other reagents, process and work-up is analogous to the protocol for the classical CyD polymers (recovered solid ∼ 200 g). Based on 1H NMR spectroscopy the CyD content in the polymers is 60–70% w/w, and the polymers have a molecular weight of 50–60 kDa determined by means of size exclusion chromatography thus featuring more than 25 macrocyles per polymer.

Noticeably the synthetic protocols affording batches of more than 200 g of polymer comply with many working principles in Green Chemistry: synthesis is performed in a safe solvent, only two reagents or three, in the case of mixed polymers, are required and no catalyst, and heating up to low temperatures is sufficient to perform the polycondensation reaction. Inexpensive reagents that are produced on industrial scale were used to prepare the polymers which makes them very appealing.

### Drug encapsulation

First, a test on the amount of drug that can be dissolved by the various carriers consisting of polymers containing either one or two types of CyD was performed. We used the polymers pαCyD pβCyD and pγCyD as well as the new polymers with mixtures of two CyDs, pαβCyD, pαγCyD and pβγCyD. Optical spectroscopy was used to ascertain drug dissolution and concentration.

CBX has a low solubility in water of 15 μM. The molecule is endowed with an absorption spectrum displaying two peaks centered at 275 and 232 nm in the UV, has an intense structured circular dichroism (CD) spectrum peaking in negative at 295 nm and in positive at 230 nm being a chiral substance with different stereocenters and displays no fluorescence in water. Upon dissolution with the carrier solution the drug CD signal is not changing, and the drug does not become fluorescent. Consequently, the latter technique has not further been exploited for studying CBX. BIC has a solubility of 10 μM in water. It is endowed with an absorption spectrum with a peak in the UV at 275 nm and displays weak fluorescence with maximum at 325 nm in water. Even though BIC has one stereocenter, the solution lacks a CD spectrum as we are dealing with a racemic mixture. The large Stokes shift of circa 45 nm between the maxima in the absorption and fluorescence spectrum in water indicates important conformational differences between the ground state and emissive state. The fluorescence lifetime of BIC in water is below the instrumental limit of 300 ps. Upon dissolution by the carrier the drug acquires a CD signal, dependent in shape and intensity on the CyD nature and becomes more fluorescent. The drug displays a biexponential decay in the presence of all carriers, with a short component dominating, that equals the lower temporal resolution limit of the TCSPC instrumentation and a longer component of few ns accounting for less than 10% of the emitted photons (Table S1†).

Fig. 1 shows the absorption, and CD spectra of the drugs dissolved with 10 mg ml^−1^ carrier preparing films for 60 μM drug concentration. Considering 60% w/w of the CyD in the polymer, the CyD concentration is *ca* 4–6 mM range, so in terms of CyD concentration a large excess is achieved.

The absorption spectra of CBX already allow for a discrimination of the different polymers (Fig. 1a). Transparent solutions have been obtained only for pβ- and pαβCyD and all other solutions present turbidity indicative of an incomplete/partial drug dissolution in the chosen experimental conditions. Solutions of the carriers alone are totally transparent. Centrifugation allows to eliminate undissolved matter and confirms the best dissolution is obtained with pβCyD or the mixed pαβCyD. Also CD spectra confirm the same trend (Fig. 1b). Dissolving the drug either as film obtained from EtOH or as powder with the polymer solution afforded similar results. The same protocol was used to identify the best polymer to solubilize BIC. 10 mg ml^−1^ CyD polymer solutions were adopted to solubilize films for 60 μM BIC concentration obtained from evaporated EtOH solutions. Solutions were stirred for 18 h. In the case of BIC the differentiation is not emerging in terms of drug solubility as approximatively the same drug amounts are dissolved with the different carriers looking at the absorbance maxima of 270 nm band (Fig. 1c). Interestingly, the CD spectra are very different in shape and intensity depending on the polymer nature (Fig. 1d). Comparing the spectra for single CyDs with those of the mixed polymers it appears that in the mixed polymers both CyDs participate in guest binding as BIC has a spectrum in the mixed polymers resembling the sum of BIC CD signals in the single polymers. As to the fluorescence of the drug strong variations in intensity are observed for the different polymers (Fig. S2a†) and the lifetime is still very short with a value of 0.35 ns not varying with the polymer and a longer component 2–3 ns that contributes very marginally to the total decay (Table S1†). All together these data indicate the drug experiences a varying local environment in the different polymeric systems.

We performed spectrophotometric experiments to determine the molar absorption coefficient of the different drugs encapsulated in the best performing polymers (See Fig. S3 in ESI†). This information is crucial to evaluate the efficiency of encapsulation of the polymers for the drugs separately and together. Low amounts of drugs in the 5–60 μM range were prepared as films from EtOH and dissolved with fixed amount of polymer, 10 mg ml^−1^. After subtraction of the CyD contribution, plotting the absorbance *vs.* the drug concentration always afforded an excellent linear plot with the slope giving the molar absorption coefficient reported in Table 1 for the different systems. The same linearity has been obtained for the ellipticity at selected wavelengths plotted *vs.* the drug concentration (see Fig. S3 in ESI†). The *ε* values were used to calculate the amount of dissolved drug in all experiments. Only small changes are observed in the absolute values of the maximum absorbance wavelength and the molar absorption coefficients for the drugs in the different polymers.

**Table 1:** Molar absorption coefficients of the drugs complexed to the polymers^a^

	CBX		BIC	
	*λ*_max_ (nm)	*ε* (M^−1^ cm^−1^)	*λ*_max_ (nm)	*ε* (M^−1^ cm^−1^)
pαCyD	—	—	271	19 280 ± 160
pβCyD	232	14 995 ± 370	275	18 920 ± 120
pαβCyD	232	15 100 ± 72	274	18 930 ± 50

a Empty pCyD absorbance signal has been subtracted from all curves.

Using optical spectroscopy, we determined the equilibrium binding constants of the drugs with the best performing polymers by titrating solutions with low drug concentration, kept below its solubility, with increasing pCyD amounts. Global multiwavelength analysis using the Reactlab™ Equilibria software was performed on a set of spectra obtained for mixtures with fixed drug concentration and increasing polymer amounts. In the case of BIC we used the fluorescence spectra strongly changing in intensity upon polymer addition (Fig. 2a and S4†). For CBX we used absorption spectra for the global analysis as CD of CBX was not changing significantly enough to afford reliable analysis. Fig. 2b shows the spectra used for the calculation of the binding constants. In order to fit the data we explored several binding models with either one or two complexes having different stoichiometry, and we used the CyD concentration and not the polymer concentration. In the case of BIC, the fit converges with a model with one complex of 1 : 2 (drug : CyD) stoichiometry and the binding constants do not differ for the 3 polymers examined and (see Table 2). This is a plausible stoichiometry considering the structure of the drug with two aromatic rings that can fit each one CyD cavity. Fig. S4c–e† shows graphs illustrating the goodness of the fit by means of a comparison of the experimental and calculated values at selected wavelengths, the distribution of the various species *vs.* total bCyD concentration and the calculated spectra of free and complexed BIC. Affinity of CBX is higher for the polymer and also in this case two CyD units are complexing the drug in the polymer. The binding constants for BIC and CBX are also significantly higher compared to previous binding constants obtained with the same polymer for the antibiotic ethionamide and a synthetic booster with logK_1:1_ of 1.9 and 2.9,^34^ respectively, as well as for the antitumoral Doxorubicin with log *K*_1:1_ of 2.2.^51^

**Table 2:** Binding constants for the model with two complexes obtained with global analysis of the spectra with the Software Reactlab™ Equilibria

	log *K*_1:2_/M^−2^	log *K*_1:1_/M^−1^	Technique
BIC + pαCyD	6.8		Fluorescence
BIC + pβCyD	6.9		Fluorescence
BIC + pαβCyD	7.4		Fluorescence
CBX + pβCyD	10.2		Absorption
Ce6 + pβCyD	4.0	2.9	Absorption
Ce6 + pβCyD	4.1	3.0	Fluorescence

We explored if the dissolution time or the amount of solid drug influences the maximum amount of solubilized drug (Fig. S1 and S2b in ESI†). Two different amounts of solid BIC were tested and dissolved with aqueous polymer solutions and the results in Table 3 show that high drug amounts do not favour increased solubility. This holds particular for pαCyD and pαβCyD. Only for pβCyD we observe improved solubility with the high amount of solid drug. As concerns the time interval applied to dissolve the solid drug 36 h yielded better results compared to 5 days dissolution time.

**Table 3:** Concentration^a^ of BIC dissolved in water solutions of 10 mg ml^−1^ polymer under different conditions

	[BIC]/M				
	0.026 mg ml^−1^ (0.6 × 10^−4^ M)			0.075 mg ml^−1^ (1.8 × 10^−4^ M)	
	18 h	36 h	5 days	36 h	5 days
pαCyD + BCA	0.51 × 10^−4^	0.43 × 10^−4^	0.20 × 10^−4^	0.23 × 10^−4^	0.20 × 10^−4^
pβCyD + BCA	0.54 × 10^−4^	0.61 × 10^−4^	0.62 × 10^−4^	1.0 × 10^−4^	0.65 × 10^−4^
pαβCyD + BCA	0.49 × 10^−4^	0.62 × 10^−4^	0.61 × 10^−4^	0.53 × 10^−4^	0.39 × 10^−4^

a Values calculated with the molar absorption coefficients in Table 1.

The same tests have been performed also for CBX (Table 4). Again, the increase in dissolution time and solid drug amount does not improve the dissolution capacity of the selected polymers. Overall for both drugs the best results were obtained with the pβCyD and a dissolution time of 36 h. Considering the solubility of 15 μM and 10 μM, respectively, of CBX and BIC alone in water, the solubility with the polymers present in 10 mg ml^−1^ is increased up to 10 times.

**Table 4:** Concentration^a^ of CBX dissolved in water solutions of 10 mg ml^−1^ polymer under different conditions

	[CBX]/M			
	0.034 mg ml^−1^ (0.4 × 10^−4^ M)		0.086 mg ml^−1^ (1 × 10^−4^ M)	
	36 h	5 days	36 h	5 days
pαCyD + CBX	0.39 × 10^−4^	0.37 × 10^−4^	0.45 × 10^−4^	0.34 × 10^−4^
pβCyD + CBX	1.0 × 10^−4^	1.0 × 10^−4^	0.91 × 10^−4^	0.88 × 10^−4^
pαβCyD + CBX	0.74 × 10^−4^	0.60 × 10^−4^	0.70 × 10^−4^	0.72 × 10^−4^

a Values calculated with the molar absorption coefficients in Table 1.

### Computational study of the drug βCyD interaction

Being the polymer a large molecule the computational DFT study has been conducted on a model consisting of the drug encapsulated by either 1 or 2βCyD units chosen as the pβCyD seems the most promising for the drug delivery purpose. In previous works, DFT methods have been extensively used to study the inclusion process of drugs in βCyD,^52–56^ with the inclusion of the dispersion correction method.^57^ In these works, calculations of the interaction energy (IE), binding free energy, natural bond orbital (NBO) and natural population analysis (NPA) were used to investigate the inclusion process, while the non-covalent interaction method (NCI)^58^ or independent gradient model (IGM) analysis^49^ were employed to study in-depth the non-covalent interaction in the host guest complex.

#### 1 : 1 stoichiometry model

**Fig. 3:**
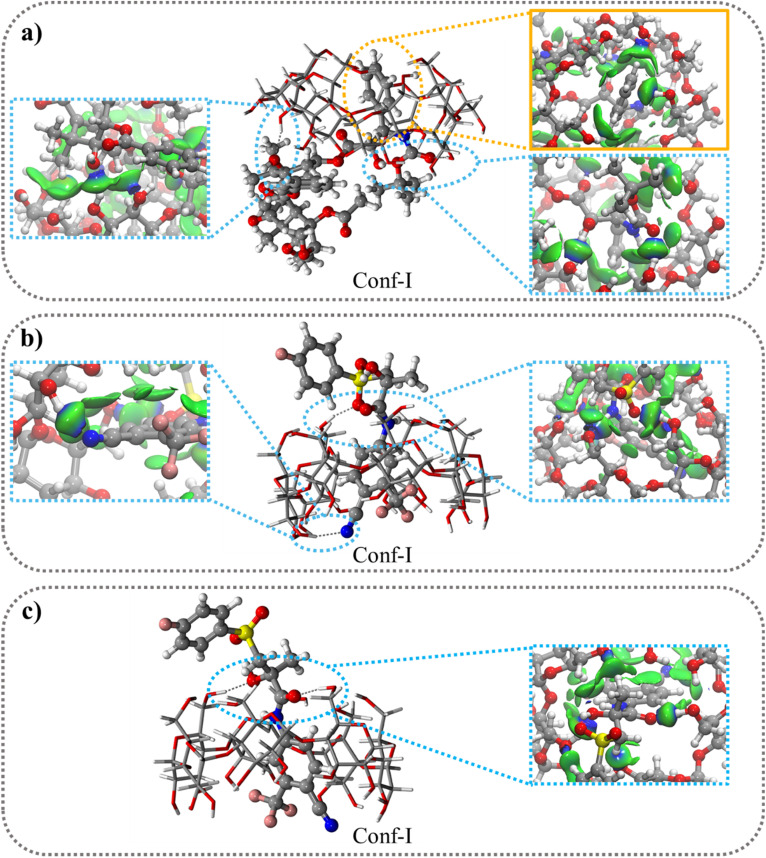
Optimized most stable conformation for 1 : 1 stoichiometry (Conf I) of βCyD and: (a) CBX; (b) S-BIC; (c) R–BIC; with color-filled δ*g*^inter^(*ρ*) projection (isovalue 0.0055 a.u.). vdW and HBs interactions are highlighted by yellow and blue circle respectively.

Both BIC and CBX contain two hydrophobic aromatic rings and two conformations, Conf-I and Conf-II, (Fig. S5†) were studied differing for the aromatic ring included in the βCyD host. The optimized structures of the 1 : 1 complexes are shown in Fig. 3 and S6† for Conf-I and Conf-II, respectively. All complexes are stabilized in energy with respect to the separate reactants as highlighted by the negative IE values in Table 5 ranging from −26.9 kcal mol^−1^ for Conf-II R–BIC@βCyD to −41.9 kcal mol^−1^ for Conf-I CBX@βCyD. In all cases Conf-I complexes are more stable than Conf-II complexes and follow the order: CBX@βCyD > S-BIC@βCyD > R-BIC@βCyD. Interestingly, S-BIC presents slightly stronger interaction with βCyD in Conf-I compared to R-BIC with a difference IE, ΔIE_S-BIC-R-BIC_, of −5.2 kcal mol^−1^ likely due to the different orientation of the SO_2_ group (Fig. 3b and c).

**Table 5:** Interaction energy (IE), total deformation energy (Def) and deformation energy for βCyD (Def_βCyD_) and guest molecules (Def_guest_) for inclusion complexes investigated in water (kcal mol^−1^). IE and Def for the interaction of the 2 βCyD without guest molecules

Complexes	IE	Def	Def_βCyD_	Def_guest_
**1 : 1 stoichiometry**				
βCyD-CBX Conf-I	−41.9	29.7	15.1	14.6
βCyD-CBX Conf-II	−35.0	16.2	11.9	4.3
βCyD-S-BIC Conf-I	−35.1	12.6	5.7	6.9
βCyD-S-BIC Conf-II	−27.5	10.3	3.9	6.4
βCyD-R-BIC Conf-I	−29.9	8.5	5.3	3.2
βCyD-R-BIC Conf-II	−26.9	18.4	1.4	17.0

**1** **:** **2 stoichiometry**				
2βCyD-CBX	−65.8	65.0	52.2	12.8
2βCyD-S-BIC	−36.3	8.0	3.8	4.2
2βCyD-R-BIC	−31.6	14.1	6.0	8.1

**Formation of 2 βCyD complex**				
2βCyD (BIC)	−79.1	20.2	10.1/10.1	
2βCyD (CBX)	−50.4	25.1	8.7/16.4	

Further, the inclusion process for CBX and both BIC enantiomers induces large geometric conformational changes in the guest molecules as highlighted by the positive values of deformation energies, Def_guest_ (Table 5 and Fig. S9†). The size of CBX leads also to important conformational changes in βCyD as shown by the host deformation energy value, Def_βCyD_, (Fig. S10†), while BIC causes limited changes likely due to its smaller size (Fig. S10†).

The 2D δ*g*^inter^(*ρ*) plot (Fig. S8†) and 3D color-filled δ*g*^inter^(*ρ*) projections (Fig. 3 and S7†) allow to visualize and estimate the weak interactions such as, van der Waals (vdW) and hydrogen bonds (HBs), playing a crucial role in the formation and stabilization of inclusion complexes. All 2D δ*g*^inter^ plots show more intense spikes for Conf-I complexes (blue circle) compared to Conf-II complexes (red triangle). The small and sharp spikes at −0.01 sign(*λ*_2_)*ρ* values indicate the presence of vdW interactions (CH⋯π, OH⋯π, CH⋯CH and CH⋯OH) quite similar in Conf I and II while the more intense spikes at −0.025 to −0.04 sign(*λ*_2_)*ρ* values refer to the formation of strong HBs especially in conf.

#### 1 : 2 stoichiometry complexes

**Fig. 4:**
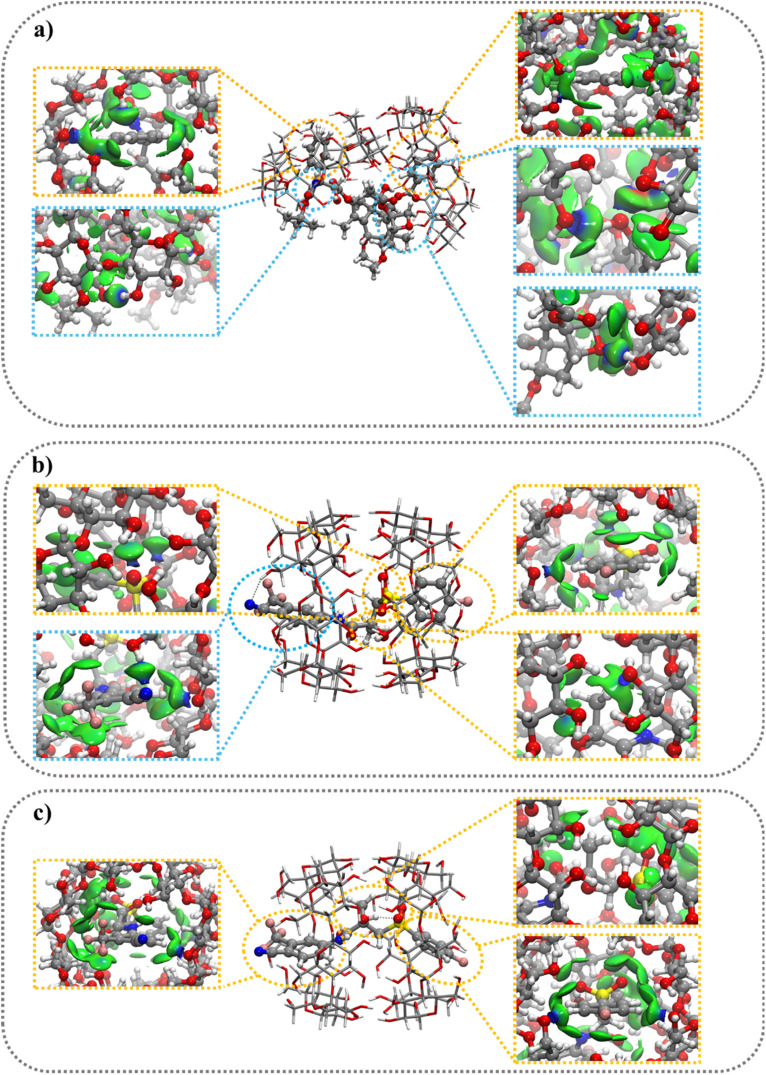
Optimized structures for 1 : 2 stoichiometry of βCyD and: (a) CBX; (b) S-BIC; (c) R–BIC; with color-filled δ*g*^inter^(*ρ*) projection (isovalue 0.0055 a.u.). vdWs and HBs interactions are highlighted by yellow and blue circle respectively.

The optimized structures of the 1 : 2 complexes show the complete inclusion of BIC molecules inside the nanocavity of two βCyDs (Fig. 4b and c). The two secondary faces of βCyDs interact with each other thanks to HBs (Fig. S12a†). A complete relaxation of this structure, without guest molecules, shows a strong IE energy of −79.1 kcal mol^−1^ (Table 5) with the two βCyD deformed in the same way. Again, CBX is partially included inside the nanocavities (Fig. 4a) and the two βCyD interact differently thanks to HBs, CH⋯O and CH⋯CH interactions between secondary faces of one βCyD and the lateral bone of the second one (Fig. S12b†). After a relaxation without guest molecule, the IE value was higher (−50.4 kcal mol^−1^) than that observed before, indicating a lower stabilization for this structure; moreover, the two bCyD show different Def_βCyD_ energies.

The more negative IE values for the 1 : 2 stoichiometry indicate that this complex is preferred over 1 : 1 complex and this is in line with the experimental findings that afford binding models with the exclusive 1 : 2 complex. The IE values range from −31.6 to −65.8 kcal mol^−1^ (Table 5) and follow the order: CBX@βCyD > S-BIC@βCyD > R-BIC@βCyD. Passing from 1 : 1 to 1 : 2 complex a decrease in IE (ΔIE_2:1 to 1:1_ = IE_2:1_ − IE_1:1_) of −23.9 kcal mol^−1^ is observed for CBX and only −1.2 and −1.7 kcal mol^−1^ for the BIC S and R enantiomer, respectively. The poor energy stabilization passing from 1 : 1 to 1 : 2 for BIC complexes, may be explained by interaction involving also the BIC unit outside the nanocavity with primary or secondary hydroxyl groups in 1 : 1 complex (Fig. 3b and c). As to the Def values a large increment is observed only for CBX@βCyD in 1 : 2 stoichiometry with a ΔDef_2:1 to 1:1_ of +35.3 kcal mol^−1^ respectively due to the huge increase of Def_βCyD_ value caused by the large distortion of the two βCyDs during the inclusion process (Fig. S13a and S14a†). No significant difference in Def_guest_ and Def_βCyD_ values was observed for the formation of the 1 : 2 complexes for BIC enantiomer (Fig. S13b, c, S14b and c†).

Differently from the 1 : 1 complex, BIC@βCyD 1 : 2 complexes were stabilized almost exclusively by weak vdW interactions as shown in Fig. 4b, c, S11b and c,† emphasizing, once again, the crucial role of this type of interactions. CBX@βCyD 1 : 2 complexes show the formation of both weak vdW and HBs interactions (Fig. 4a and S11a†), where the energy contributions of HBs were higher than those observed for vdW interactions.

### Calculated absorption and CD spectra

**Fig. 5:**
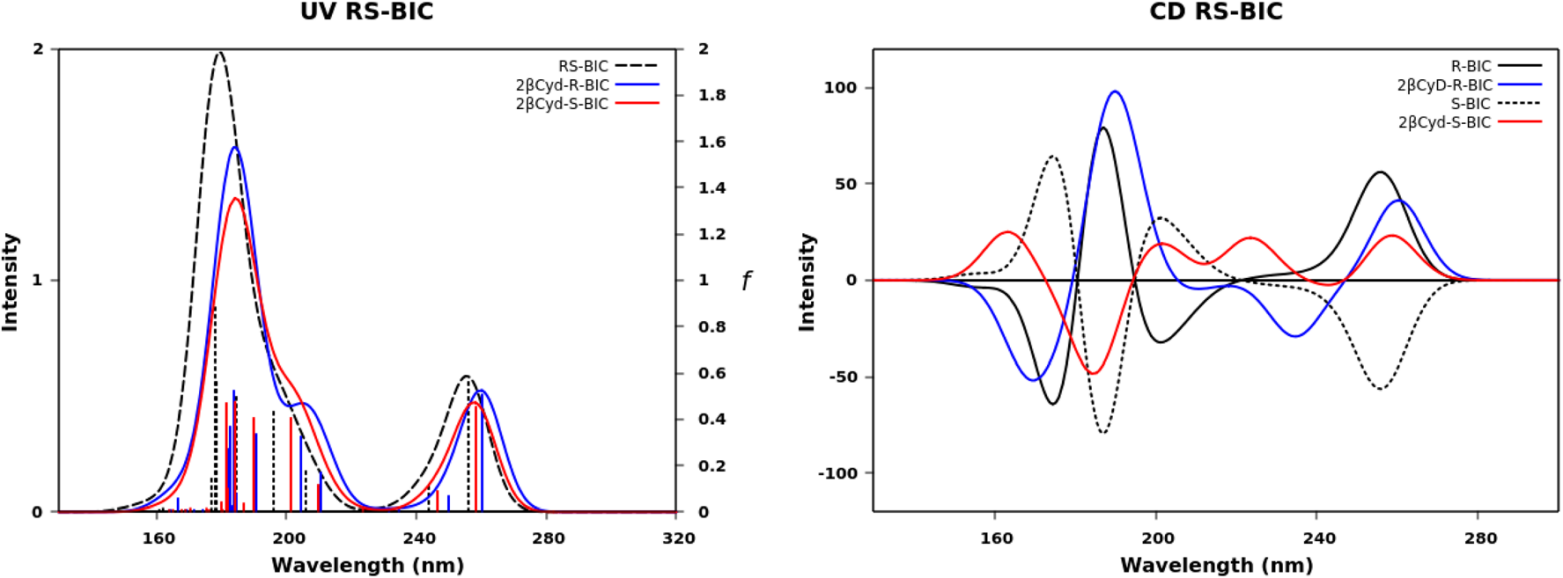
Calculated UV absorption spectra, oscillator strength *f* and CD spectra of R–BIC and S-BIC (black dashed line), 2βCyD-R-BIC (blue line) and 2-βCyD-S-BIC (red line).

We further exploited the results described above, and carried out a computational analysis of the photophysical properties of the drugs R–BIC, S-BIC, and CBX and of their corresponding 1 : 2 βCyD complexes by means of DFT and TD-DFT calculations (see experimental part). Fig. 5 shows the UV and CD spectra of R-, S-BIC, and the 1 : 2 complexes and Table S2† reports the *f* and *R* values. These spectra are slightly blue shifted compared to the experimental spectra.

**Fig. 6:**
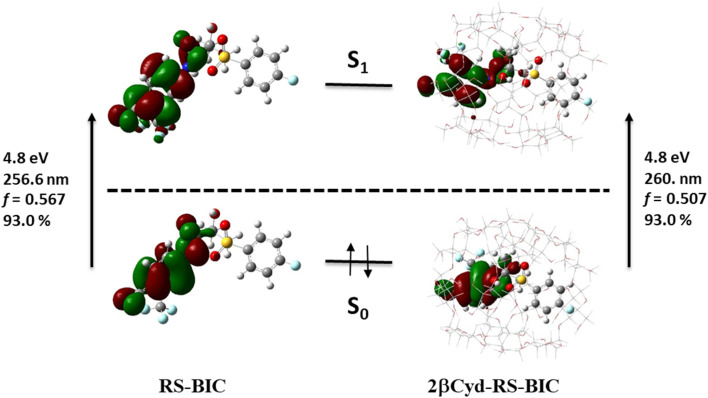
Orbitals, transition energies (nm and eV) and oscillator strength (*f*) of the lowest energy transition of RS-BIC and 2βCyD-RS-BIC.

The two identical spectra of R–BIC and S-BIC exhibit two bands in the regions 160–220 nm and 240–280 nm. The lowest transition at 256.0 nm (*f* = 0.568) is a local π → π* transition involving the cyano-(trifluoromethyl)phenyl moiety and the involved orbitals are illustrated in Fig. 6. The second transition at 243.9 nm (*f* = 0.118) is still a π → π* transition also involving the same moiety with HOMO, and HOMO-1 orbitals mixed.

The BIC@βCyD 1 : 2 complexes display very similar features except for the small red shift upon complexation that are in line with the experimental absorption spectra of BIC and the 1 : 2 complex in Fig. S3.† The CD spectrum shows a lower intensity of the 2βCyD-S-BIC slightly blue-shifted. Noticeably, both 1 : 2 complexes of BIC have the lowest energy band positive which nicely parallels the experimental spectrum. In the latter case we need to consider a sum of the R and S enantiomer as we used the racemic mixture in the experiments. Also at lower wavelengths summing the spectra of complexed R and S enantiomer we can infer that they mirror the experimental CD where the lowest energy band positive is followed by a small negative band at 250 nm and intense a positive band at 230 nm (Fig. S3a†).

**Fig. 7:**
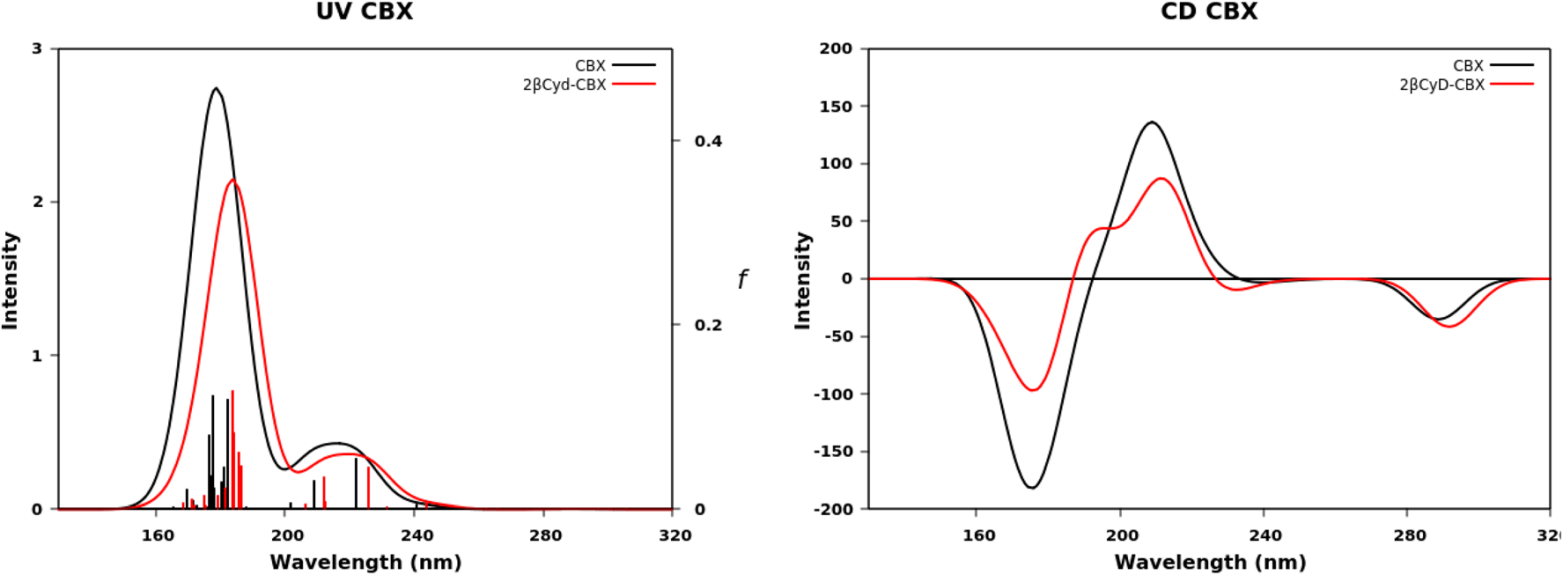
Calculated UV absorption spectra, oscillator strength *f* and CD spectra of CBX (black dashed line) and 2-βCyD-CBX (red line).

The UV spectra in Fig. 7 of CBX and CBX complex have the same characteristics apart from a small red shift (Fig. 7). Two bands are observed the first between 150 and 200 nm and the second between 200 and 240 nm. More than five transitions are responsible of the second band, but only the transition to the S_5_ has a significant oscillator strength value (see *f* values in Table S2†) and is π → π* centered on the [phenylpropanoyl]oxy moiety. The small red-shift of in the complex is due to a change in the transition to S_2_ state, also a local π → π* transition on the same moiety, displaying a larger *f* value, similar to the transition to S_5_ state. Also the experimental spectra show only small changes upon complexation (Fig. 2). The lowest transitions in the CD spectra of free and complexed CBX mirror the experimental spectra, and only in the far UV CD is less intense for the complex and a shoulder at 190 nm appears. Overall, the most stable structures represented by the 1 : 2 complexes are endowed with calculated UV and CD spectra strongly resembling the experimental spectra of the complexes and free species.

### Photosensitizer Ce6 encapsulation

**Fig. 8:**
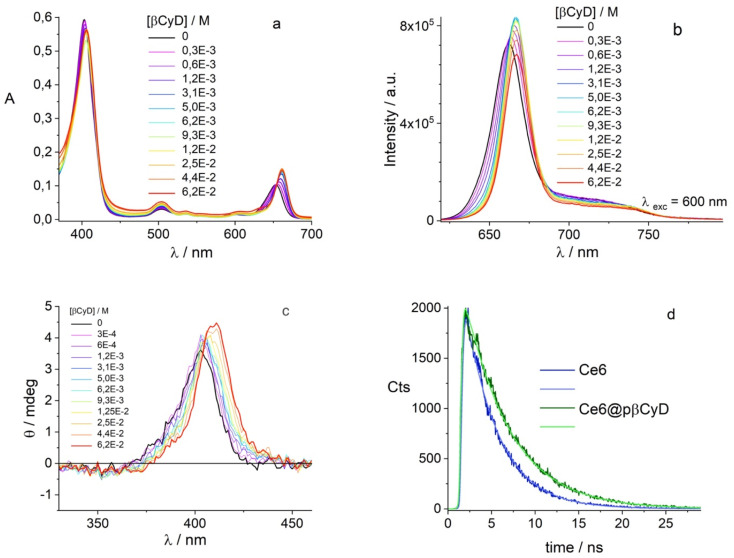
Titration study of 3.2 μM Ce6 with different amounts of pβCyD; (a) absorption spectra in 1 cm cuvet; (b) fluorescence spectra; (c) CD spectra in 1 cm cuvet; (d) Ce6 fluorescence decay with and without pβCyD.

Being our interest the implementation of the combination of chemo- and photodynamic therapy we also assessed the affinity of the PS chlorin e6 for pβCyD. As this PS is soluble in water and acts as catalyzer for ^1^O_2_ production, the capacity of the polymer to dissolve high amounts of PS has not been explored. Fig. 3 shows the absorption, fluorescence and CD spectra for mixtures of Ce6 with the most promising polymer pβCyD. In all three techniques the signals change significantly with the increasing CyD concentration, indicating complexation of Ce6 with the polymer is occurring. Also, the fluorescence lifetime changes from 3.5 to 4.7 ns performing a global analysis of different solutions of mixtures (Fig. 8d and S15†). This lifetime change represents an interesting tool to monitor the complexed state of Ce6 in biological environments such as cell cultures by means of lifetime imaging. The binding model emerging is that of two complexes with 1 : 1 and 1 : 2 stoichiometry with log *K* values of 3.0 and 4.1 respectively (Table 2). Fig. S15† shows the spectra of the various species, the species distribution and the correspondence of experimental and calculated values at selected absorption and fluorescence wavelengths. This rather low affinity is not surprising considering the rather good solubility of Ce6 in water.

In conclusion Table 2 collects the binding constants of the single components for the most promising polymers. Compared to binding constants of other drugs such as ethionamide (p*K*_1:1_ of 1.9 M^−1^), artemisinin (p*K*_1:1_ of 2.0 M^−1^) and doxorubicin (p*K*_1:1_ of 2.2 M^−1^) with the same polymer the affinity is one order of magnitude higher for BIC and Ce6 and even 3 orders for CBX.^34,51^

### Drug co-encapsulation

The next part of the work focused on the co-encapsulation of the drugs and Ce6. In the case of lower amounts of solid drug, the polymers pβCyD and pαβCyD allowed complete dissolution while the pαCyD polymer did not (Table 6). At higher amounts of solid drug we discern a discrimination also between pβCyD and pαβCyD. We can observe a positive effect on the encapsulated quantity of BIC in the co-loading for all three polymers; note that lower amounts of CBX are dissolved, but this drug is anyhow needed in lower amounts *in vivo*. Overall, the pβCyD is the best polymer choice among those tested as it loads the highest amounts of a combination of two drugs. Similar data have been obtained for the pβCyD in PBS solutions. Noticeably up to 0.1 mM drug can be dissolved with 10 mg ml^−1^ of polymer corresponding to 4–6 mM CyD meaning that we still have a strong excess in CyD units *vs.* drug and this amount can be further increased considering the solubility of the polymer up to 200 mg ml^−1^. Considering the concentrations reported in ref. 35 and 36 we prepared solutions containing 100 mg ml^−1^ of pβCyD loading 1.7 mM of BIC and 10 nM of CBX, required in much lower amount due to its low IC50 value, confirming our hypothesis on the polymer ability to dissolve higher drug amounts.

**Table 6:** Concentration^a^ of dissolved CBX and BIC with 10 mg ml^−1^ polymer in water starting from different solid drug amounts^b^

	[CBX] and [BCA] (M)		[CBX] and [BCA] (M)	
**Solid drug amount**	**1.0 × 10^−^** ^**4**^ **CBX**	**0.6 × 10^−^** ^**4**^ **BIC**	**2.3 × 10^−^** ^**4**^ **CBX**	**1.7 × 10^−^** ^**4**^ **BIC**
pαCyD + CBX	0.32 × 10^−4^		0.32 × 10^−4^	
pαCyD + BIC		0.33 × 10^−4^		0.19 × 10^−4^
pαCyD + BIC + CBX	0.36 × 10^−4^	0.63 × 10^−4^	0.32 × 10^−4^	0.36 × 10^−4^
pβCyD + CBX	1.0 × 10^−4^		0.79 × 10^−4^	
pβCyD + BIC		0.63 × 10^−4^		1.10 × 10^−4^
pβCyD BIC + CBX +	1.0 × 10^−4^	0.63 × 10^−4^	0.71 × 10^−4^	1.67 × 10^−4^

**Solid drug amount**	**0.8 × 10^−^** ^**4**^	**0.4 × 10^−^** ^**4**^	**1.9 × 10^−^** ^**4**^	**1.50 × 10^−^** ^**4**^
pαβCyD + CBX	0.82 × 10^−4^		0.60 × 10^−4^	
pαβCyD + BIC		0.41 × 10^−4^		0.64 × 10^−4^
pαβCyD + BIC + CBX	0.72 × 10^−4^	0.36 × 10^−4^	0.56 × 10^−4^	1.44 × 10^−4^

a Values calculated according to the method described in the experimental part.

b 36 h of stirring.

We used DLS to investigate the dimension of unloaded and loaded polymers assembling in particles with a size below 20 nm. Table 7 shows the most representative data. The polymeric CyD NPs not only have a very small hydrodynamic diameter, but they have a refractive index very similar to the solvent (water), therefore the measurement of their size by means of DLS was particularly challenging. The analysis confirmed the small dimension of the polymers organized in NPs with the drug presence not significantly affecting their dimension.

**Table 7:** DLS data for the loaded polymers

Sample	Peak 1 (nm)	Peak 2 (nm)	Peak 3 (nm)	Peak 1 (%)
pαCyD	13	300	3000	87
pβCyD	20	—	4000	98
pαβCyD	12	400	5000	88
pαCyD + CBX	14	250	—	78
pαCyD + BCA	14	250	5000	80
pβCyD + CBX	21	500	4000	90
pβCyD + BCA	15	150	—	84
pαβC_y_D + CBX	11	230	5000	82
pαβCyD + BCA	11	150	5000	85
pαCyD + CBX + BCA	11	300	800	49
pβCyD + CBX + BCA	19	500	—	81
pαβCyD + CBX + BCA	11	250	5000	63

In conclusion, the most promising polymers are pβCyD and pαβCyD as they dissolve the highest amount of BIC, and CBX or their combination and drug loaded polymer solutions have satisfactory stability profile over 28 days (Fig. S16†). The data obtained evidence that the CyD cavity is playing a role but this cannot be explained solely with the size of the drug and the cavity. As expected, polymers with αCyD behave worse for CBX, a very large molecule compared to the cavity. However, the polymers with γCyD resulted not to be efficient in solubilizing CBX in spite of their large cavity. In the case of BIC a similar situation holds and polymers with βCyD outstand; CD spectra show that there is likely no good interaction with the αCyD as the drug is not acquiring a significant signal differently from the other polymers.

**Fig. 9:**
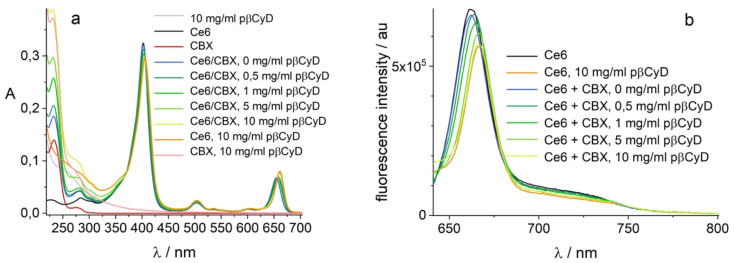
Co-encapsulation of 2 μM Ce6 and 20 μM CBX in pβCyD; (a) absorption in 1 cm; (b) fluorescence *λ*_exc_ = 610 nm.

We checked also the co-encapsulation of the chemotherapeutic drug CBX (20 μM) and the PS (2 μM). Polymer was added in increasing amounts. Both compounds become encapsulated with largest amount of CBX dissolved with 10 mg ml^−1^ polymer (Fig. 9) and Ce6 exhibiting a mono-exponential decay with lifetime of 5.1 ns, assigned of a complexed form. Global fluorescence lifetime analysis confirms increasing amounts of polymer shift the complexation equilibrium of Ce6 to complexed form (Fig. S17†). We can conclude that co-encapsulation is possible for both compounds and Ce6 photophysical behavior is not altered by the drug.

**Fig. 10:**
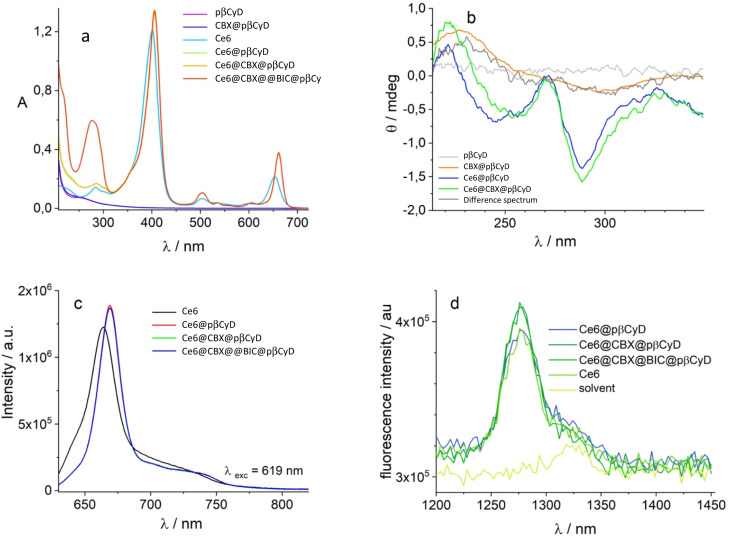
Co-encapsulation of 4 μM of CBX, 200 μM of BIC and 50 μM of Ce6 in 50 mg ml^−1^ pβCyD solution; (a) absorption and (b) CD spectra; (c) co-encapsulation of 0.1 μM of CBX, 100 μM of BIC and 50 μM of Ce6 in 10 mg ml^−1^ pβCyD solution: fluorescence of Ce6 (black), Ce6@pβCyD (red); Ce6@CBX@pβCyD (green); Ce6@CBX@BIC@pβCyD (blue) and (d) ^1^O_2_ emission.

The last part of the study concerned the co-encapsulation of Ce6, BIC and CBX. We used the above discussed spectroscopic techniques to evaluate the complexation of the 3 components in pβCyD. Ce6 complexation is unambiguously evidenced by the shift of the Soret band and the peak at 650 nm in the absorption spectra as well as the shift of the Soret band in the CD spectra (Fig. 10). Also fluorescence confirms the complexed state of the PS as well as the single lifetime of 5.0 ns in all polymer solutions containing Ce6. A low concentration of CBX has been chosen for this experiment as this drug eventually needs to be administered in much lower concentrations considering its IC50 in the nM range for the prostate cancer cell line. The presence of 0.1 μM CBX remains to be confirmed as the signals of CBX both in absorption and CD are covered by the signals of the other two components. After subtraction of the Ce6 signal, 100 μM concentration of BIC is confirmed using the molar absorption coefficients of 18 920 M^−1^ cm^−1^ at 275 nm. The changes in the UV range comparing the spectra of pβCyD/Ce6 with the spectra of pβCyD/Ce6/BIC confirmed the complexed state of the drug.

In an attempt to ascertain the CBX complexation the conditions for co-encapsulation of the three compounds were changed. We increased the amount of polymer to 50 mg ml^−1^ and dissolved 4 μM of CBX, 200 μM of BIC and 50 μM of Ce6 PS. Subtracting the polymer/Ce6 spectrum from the polymer/Ce6/CBX spectrum we can discern the CD spectra typical of CBX in the polymer (Fig. 10b). Most importantly, Ce6 also in the presence of the two drugs produces singlet oxygen with the same efficiency compared to Ce6 in water (Fig. 10).

## Conclusions

In this work we explored a series of CyD polymers composed either of a single CyD type or a mixture of two CyD types for the encapsulation of different therapeutic compounds. Spectroscopic techniques allowed to establish unequivocally the encapsulation of the 3 compounds in the polymeric systems: two drugs, a taxane and androgen receptor, and a photosensitizer. The polymers pβCyD and pαβCyD had the best performance in terms of separate drug solubilization. Studying the co-encapsulation of the two drugs pβCyD and pαβCyD differentiate in drug solubilization ability with pβCyD performing best. Avoiding the use of organic solvents it was possible to dissolve up to 0.1 mM CBX and BIC with 10 mg ml^−1^ βCyD polymer and, with 100 mg ml^−1^ polymer, even 1.7 mM for BIC, a 100-fold improvement compared to their solubility in water. Spectroscopic studies in the presence of the βCyD polymer afforded the binding constants for the 1 : 2 complex of CBX and BIC with CBX displaying higher affinity. Both experimental data and DFT calculations suggested that the drugs are complexed by two CyD units in the polymer. The PS Ce6 has also good affinity for the βCyD polymer and could be encapsulated simultaneously with the other two drugs. Most importantly the PS is able to produce singlet oxygen also in the presence of high drug amounts. Thanks to a single inexpensive CyD-based polymer we were able to produce a three-in-one platform for future implementation of multimodal cancer therapy. These achievements are most relevant as nanomedicines are continuously proposed but their potential for translation to the pharma industry is compromised by their limited potential for industrial upscale. The performance of the system will now be further investigated in *in vitro* tests on PC cell lines to assess the synergic action of the separate components as well as the light-induced cytotoxicity.

## Conflicts of interest

The authors declare no conflicts of interest.

## Supplementary Material

RA-013-D3RA01782F-s001

## References

[cit1] Data from the WHO website , GCD Cancer Today, https://gco.iarc.fr/today/home

[cit2] Yu E. M., Aragon-Ching J. B. (2022). Advances with androgen deprivation therapy for prostate cancer. Expert Opin. Pharmacother..

[cit3] Perera M., Roberts M. J., Klotz L., Higano C. S., Papa N., Sengupta S., Bolton D., Lawrentschuk N. (2020). Intermittent versus continuous androgen deprivation therapy for advanced prostate cancer. Nat. Rev. Urol..

[cit4] Shiota M., Eto M. (2016). Current status of primary pharmacotherapy and future perspectives toward upfront therapy for metastatic hormone-sensitive prostate cancer. Int. J. Urol..

[cit5] Galletti G., Leach B. I., Lam L., Tagawa S. T. (2017). Mechanisms of resistance to systemic therapy in metastatic castration-resistant prostate cancer. Cancer Treat. Rev..

[cit6] Watson P. A., Arora V. K., Sawyers C. L. (2015). Emerging mechanisms of resistance to androgen receptor inhibitors in prostate cancer. Nat. Rev. Cancer.

[cit7] Upadhyay T. K., Ali M. I., Khan F., Goel H., Mathur M., Goyal K., Moin S., Pandey P., Tanwar P., Sharangi A. B., Gautam S. D. C., Kapdi J. K., Patel K. I., Patel M. V., Parmar A. M., Kamal M. A. (2022). Nanoparticles Mediated Target-specific Drug Delivery in Prostate Cancer: An In-depth Review. Curr. Med. Chem..

[cit8] Vicente-Ruiz S., Serrano-Marti A., Arminan A., Vicent M. J. (2021). Nanomedicine for the Treatment of Advanced Prostate Cancer. Adv. Ther..

[cit9] Chen Y., Pan Y., Hu D. R., Peng J. R., Hao Y., Pan M., Yuan L. P., Yu Y. Y., Qian Z. Y. (2021). Recent progress in nanoformulations of cabazitaxel. Biomed. Mater..

[cit10] Imran M., Saleem S., Chaudhuri A., Ali J., Baboota S. (2020). Docetaxel: An update on its molecular mechanisms, therapeutic trajectory and nanotechnology in the treatment of breast, lung and prostate cancer. J. Drug Delivery Sci. Technol..

[cit11] Sortino S. (2012). Photoactivated nanomaterials for biomedical release applications. J. Mater. Chem..

[cit12] Mazzaglia A., Sciortino M. T., Kandoth N., Sortino S. (2012). Cyclodextrin-based nanoconstructs for photoactivated therapies. J. Drug Delivery Sci. Technol..

[cit13] Das S., Tiwari M., Mondal D., Sahoo B. R., Tiwari D. K. (2020). Growing tool-kit of photosensitizers for clinical and non-clinical applications. J. Mater. Chem. B.

[cit14] Zhou Z. J., Song J. B., Nie L. M., Chen X. Y. (2016). Reactive oxygen species generating systems meeting challenges of photodynamic cancer therapy. Chem. Soc. Rev..

[cit15] Obaid G., Broekgaarden M., Bulin A. L., Huang H. C., Kuriakose J., Liu J., Hasan T. (2016). Photonanomedicine: a convergence of photodynamic therapy and nanotechnology. Nanoscale.

[cit16] Zi Y. X., Yang K. Y., He J. H., Wu Z. M., Liu J. P., Zhang W. L. (2022). Strategies to enhance drug delivery to solid tumors by harnessing the EPR effects and alternative targeting mechanisms. Adv. Drug Delivery Rev..

[cit17] Pei Z. R., Chen S. T., Ding L. Q., Liu J. B., Cui X. Y., Li F. Y., Qiu F. (2022). Current perspectives and trend of nanomedicine in cancer: A review and bibliometric analysis. J. Controlled Release.

[cit18] Jiang Y. K., Jiang Z. Y., Wang M. Z., Ma L. (2022). Current understandings and clinical translation of nanomedicines for breast cancer therapy. Adv. Drug Delivery Rev..

[cit19] Dordevic S., Gonzalez M. M., Conejos-Sanchez I., Carreira B., Pozzi S., Acurcio R. C., Satchi-Fainaro R., Florindo H. F., Vicent M. J. (2022). Current hurdles to the translation of nanomedicines from bench to the clinic. Drug Delivery Transl. Res..

[cit20] Duran-Lobato M., Lopez-Estevez A. M., Cordeiro A. S., Dacoba T. G., Crecente-Campo J., Torres D., Alonso M. J. (2021). Nanotechnologies for the delivery of biologicals: Historical perspective and current landscape. Adv. Drug Delivery Rev..

[cit21] Kurkov S. V., Loftsson T. (2013). Cyclodextrins. Int. J. Pharm..

[cit22] Renard E., Deratani A., Volet G., Sebille B. (1997). Preparation and characterization of water soluble high molecular weight β-cyclodextrin-epichlorohydrin polymers. Eur. Polym. J..

[cit23] Malanga M., Balint M., Puskas I., Tuza K., Sohajda T., Jicsinszky L., Szente L., Fenyvesi E. (2014). Synthetic strategies for the fluorescent labeling of epichlorohydrin-branched cyclodextrin polymers. Beilstein J. Org. Chem..

[cit24] Morin-Crini N., Fourmentin S., Fenyvesi E., Lichtfouse E., Torri G., Fourmentin M., Crini G. (2021). 130 years of cyclodextrin discovery for health, food, agriculture, and the industry: a review. Environ. Chem. Lett..

[cit25] Crini G., Fourmentin S., Fenyvesi E., Torri G., Fourmentin M., Morin-Crini N. (2018). Cyclodextrins, from molecules to applications. Environ. Chem. Lett..

[cit26] Agnes M., Pancani E., Malanga M., Fenyvesi E., Manet I. (2022). Implementation of Water-Soluble Cyclodextrin-Based Polymers in Biomedical Applications: How Far Are We?. Macromol. Biosci..

[cit27] Duchene D., Gref R. (2016). Small is beautiful: Surprising nanoparticles. Int. J. Pharm..

[cit28] Duchene D., Cavalli R., Gref R. (2016). Cyclodextrin-based Polymeric Nanoparticles as Efficient Carriers for Anticancer Drugs. Curr. Pharm. Biotechnol..

[cit29] Loftsson T. (2014). Self-assembled cyclodextrin nanoparticles and drug delivery. J. Inclusion Phenom. Macrocyclic Chem..

[cit30] Fulop Z., Kurkov S. V., Nielsen T. T., Larsen K. L., Loftsson T. (2012). Self-assembly of cyclodextrins: formation of cyclodextrin polymer based nanoparticles. J. Drug Delivery Sci. Technol..

[cit31] Wankar J., Salzano G., Pancani E., Benkovics G., Malanga M., Manoli F., Gref R., Fenyvesi E., Manet I. (2017). Efficient loading of ethionamide in cyclodextrin-based carriers offers enhanced solubility and inhibition of drug crystallization. Int. J. Pharm..

[cit32] Haimhoffer A., Rusznyak A., Reti-Nagy K., Vasvari G., Varadi J., Vecsernyes M., Bacskay I., Feher P., Ujhelyi Z., Fenyvesi F. (2019). Cyclodextrins in Drug Delivery Systems and Their Effects on Biological Barriers. Sci. Pharm..

[cit33] Fraix A., Kandoth N., Manet I., Cardile V., Graziano A. C. E., Gref R., Sortino S. (2013). An engineered nanoplatform for bimodal anticancer phototherapy with dual-color fluorescence detection of sensitizers. Chem. Commun..

[cit34] Salzano G., Wankar J., Ottani S., Villemagne B., Baulard A. R., Willand N., Brodin P., Manet I., Gref R. (2017). Cyclodextrin-based nanocarriers containing a synergic drug combination: A potential formulation for pulmonary administration of antitubercular drugs. Int. J. Pharm..

[cit35] Ylitalo E. B., Thysell E., Thellenberg-Karlsson C., Lundholm M., Widmark A., Bergh A., Josefsson A., Brattsand M., Wikstrom P. (2020). Marked response to cabazitaxel in prostate cancer xenografts expressing androgen receptor variant 7 and reversion of acquired resistance by anti-androgens. Prostate.

[cit36] Lombard A. P., Liu C. F., Armstrong C. M., Cucchiara V., Gu X. W., Lou W., Evans C. P., Gao A. C. (2017). ABCB1 Mediates Cabazitaxel-Docetaxel Cross-Resistance in Advanced Prostate Cancer. Mol. Cancer Ther..

[cit37] Belhocine Y., Rahali S., Allal H., Assaba I. M., Ghoniem M. G., Ali F. A. M. (2021). A Dispersion Corrected DFT Investigation of the Inclusion Complexation of Dexamethasone with beta-Cyclodextrin and Molecular Docking Study of Its Potential Activity against COVID-19. Molecules.

[cit38] Neese F. (2022). Software update: The ORCA program system-Version 5.0. Wiley Interdiscip. Rev.: Comput. Mol. Sci..

[cit39] Ernzerhof M., Scuseria G. E. (1999). Assessment of the Perdew-Burke-Ernzerhof exchange-correlation functional. J. Chem. Phys..

[cit40] Weigend F. (2006). Accurate Coulomb-fitting basis sets for H to Rn. Phys. Chem. Chem. Phys..

[cit41] Weigend F., Ahlrichs R. (2005). Balanced basis sets of split valence, triple zeta valence and quadruple zeta valence quality for H to Rn: Design and assessment of accuracy. Phys. Chem. Chem. Phys..

[cit42] Tsuzuki S., Uchimaru T. (2020). Accuracy of intermolecular interaction energies, particularly those of hetero-atom containing molecules obtained by DFT calculations with Grimme's D2, D3 and D3BJ dispersion corrections. Phys. Chem. Chem. Phys..

[cit43] Lovison D., Alessi D., Allegri L., Baldan F., Ballico M., Damante G., Galasso M., Guardavaccaro D., Ruggieri S., Melchior A., Veclani D., Nardon C., Baratta W. (2022). Enantioselective Cytotoxicity of Chiral Diphosphine Ruthenium(II) Complexes Against Cancer Cells. Chem.–Eur. J..

[cit44] Veclani D., Tolazzi M., Ceron-Carrasco J. P., Melchior A. (2021). Intercalation Ability of Novel Monofunctional Platinum Anticancer Drugs: A Key Step in Their Biological Action. J. Chem. Inf. Model..

[cit45] Dehghani A., Bahlakeh G., Ramezanzadeh B., Mofidabadi A. H. J. (2021). 2D reduced-graphene oxide (rGO) nanosheets decorated with L-histidine loaded-beta-cyclodextrin for efficient epoxy nano-composite anti-corrosion properties; DFT-D modeling/experimental assessments. FlatChem.

[cit46] Takano Y., Houk K. N. (2005). Benchmarking the conductor-like polarizable continuum model (CPCM) for aqueous solvation free energies of neutral and ionic organic molecules. J. Chem. Theory Comput..

[cit47] Chai J. D., Head-Gordon M. (2008). Systematic optimization of long-range corrected hybrid density functionals. J. Chem. Phys..

[cit48] Boys S. F., Bernardi F. (1970). Calculation of small molecular interactions by differences of separate total energies - some procedures with reduced errors. Mol. Phys..

[cit49] Lefebvre C., Khartabil H., Boisson J. C., Contreras-Garcia J., Piquemal J. P., Henon E. (2018). The Independent Gradient Model: A New Approach for Probing Strong and Weak Interactions in Molecules from Wave Function Calculations. ChemPhysChem.

[cit50] Lefebvre C., Rubez G., Khartabil H., Boisson J. C., Contreras-Garcia J., Henon E. (2017). Accurately extracting the signature of intermolecular interactions present in the NCI plot of the reduced density gradient versus electron density. Phys. Chem. Chem. Phys..

[cit51] Anand R., Manoli F., Manet I., Daoud-Mahammed S., Agostoni V., Gref R., Monti S. (2012). b-Cyclodextrin polymer nanoparticles as carriers for doxorubicin and artemisinin: a spectroscopic and photophysical study. Photochem. Photobiol. Sci..

[cit52] Rahali S., Belhocine Y., Allal H., Bouhadiba A., Assaba I. M., Seydou M. (2022). A DFT investigation of the host-guest interactions between boron-based aromatic systems and beta-cyclodextrin. Struct. Chem..

[cit53] Jafari G., Raissi H., Shahabi M. (2022). Assessment of sulfobutylether-beta-cyclodextrin as a promising Fluorometholone molecule container: DFT, Docking, Molecular dynamics and MM-PBSA free energy calculations. Mol. Simul..

[cit54] Nora M., Ismahan L., Abdelkrim G., Mouna C., Leila N., Fatiha M., Nada B., Brahim H. (2020). Interactions in inclusion complex of beta-cyclodextrin/l-Metheonine: DFT computational studies. J. Inclusion Phenom. Macrocyclic Chem..

[cit55] Bani-Yaseen A. D. (2020). The supramolecular host-guest complexation of Vemurafenib with beta-cyclodextrin and cucurbit 7 uril as drug photoprotecting systems: A DFT/TD-DFT study. Comput. Theor. Chem..

[cit56] Belhouchet H. R., Abbaz T., Bendjedou A., Gouasmia A., Villemin D. (2022). A computational study of the inclusion of beta-cyclodextrin and nicotinic acid: DFT, DFT-D, NPA, NBO, QTAIM, and NCI-RDG studies. J. Mol. Model..

[cit57] Xu P., Alkan M., Gordon M. S. (2020). Many-Body Dispersion. Chem. Rev..

[cit58] Boto R. A., Peccati F., Laplaza R., Quan C. Y., Carbone A., Piquemal J. P., Maday Y., Contreras-Garcia J. (2020). NCIPLOT4: Fast, Robust, and Quantitative Analysis of Noncovalent Interactions. J. Chem. Theory Comput..

