# Supplementary material for: Three-in-one: exploration of co-encapsulation of cabazitaxel, bicalutamide and chlorin e6 in new mixed cyclodextrin-crosslinked polymers
Source: RSC Adv. 2023 Apr 6;13(16):10923–39. doi: 10.1039/d3ra01782f (PMC10077339; doi:10.1039/d3ra01782f)

Electronic Supplementary Material (ESI) for RSC Advances.
This journal is © The Royal Society of Chemistry 2023

Three-in-one: exploration of co-encapsulation of Cabazitaxel, Bicalutamide and Chlorin e6 in new mixed Cyclodextrin-crosslinked polymers.

Elisabetta Pancani,[3] Daniele Veclani,[1] Marco Agnes,[1] Arianna Mazza,[1] Alessandro Venturini,[1] Milo Malanga,[2] Ilse Manet[1]*

[1] Institute for Organic Synthesis and Photoreactivity, National Research Council, Via Gobetti 101, 40129 Bologna, Italy
[2] Previous affiliation: CycloLab, Cyclodextrin R&D Ltd., Budapest, Hungary
Current affiliation: CarboHyde Zrt., Budapest, Hungary
[3] Previous affiliation: Institute for Organic Synthesis and Photoreactivity, National Research Council, Via Gobetti 101, 40129 Bologna, Italy
Current affiliation: Advanced Accelerator Applications, A Novartis Company, via Ribes 5, 10010 Ivrea, Italy

Electronic Supplementary Information

1) Synthesis of the polymer

Scheme 1 below shows the reaction conditions for the synthesis of the polymers in alkaline aqueous solutions.

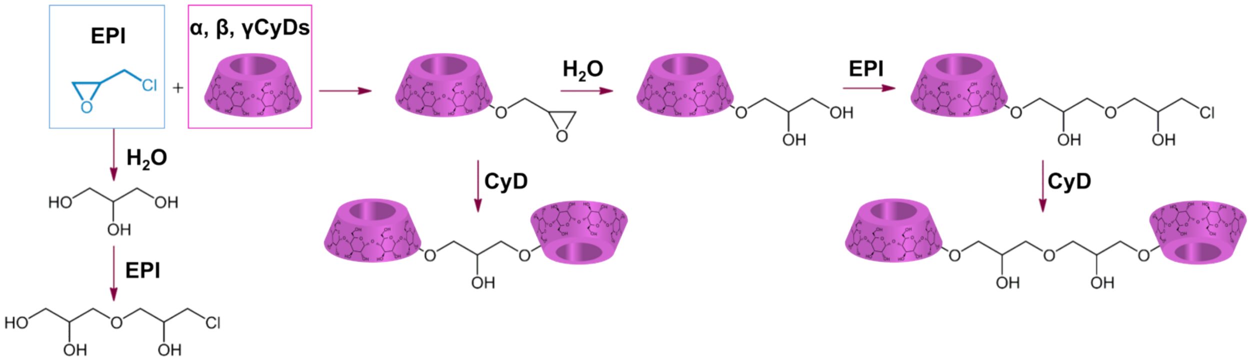

2) Solubility of CBX

Figure S1: Spectra of CBX of the dissolution experiment with different amounts of solid kept stirring for 36 hrs (solid line) and 5 days (dashes).

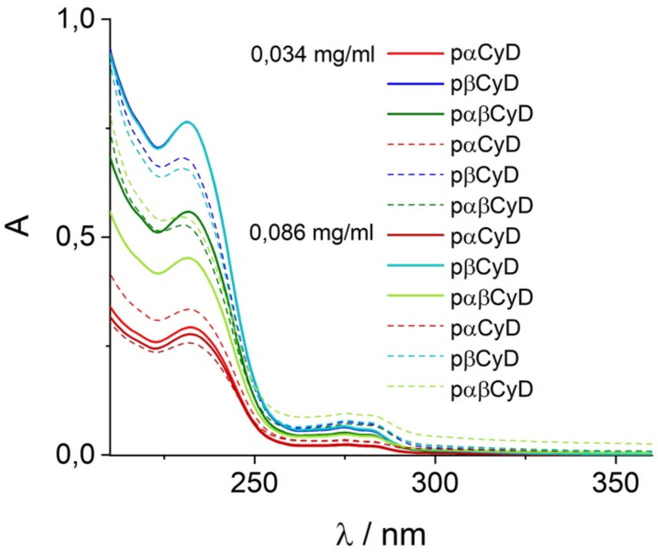

3) Solubility of BIC

Figure S2a: 10 mg/ml of polymer with 0,026 mg/ml solid drug amount; fluorescence spectra in 1 cm cuvet of 10x diluted solutions exciting at 270 nm in isosbestic point.

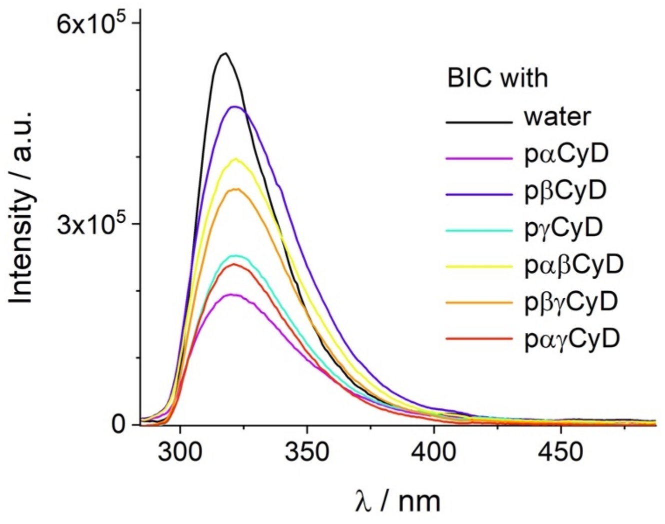

Table S1: the fluorescence lifetime, relative amplitude and average lifetime of BIC in the aqueous solutions of the different polymers

	τ_j / ns	α_j	τ_{av} / ns
**water**	0,16		
**pγCyD**	0,35 and 2,1	0,99 and 0,01	0,36
**pβγCyD**	0,36 and 1,8	0,99 and 0,01	0,38
**pαγCyD**	0,35 and 2,6	0,99 and 0,01	0,38
**pαCyD**	0,3 and 3,7	0,99 and 0,01	0,31
**pβCyD**	0,38 and 3,4	0,99 and 0,01	0,39
**pαβCyD**	0,35 and 2,8	0,99 and 0,01	0,37

Figure S2b: Absorption spectra of BIC of the dissolution experiment with different amounts of solid kept stirring for 36 hrs (solid line) and 5 days (dashes).

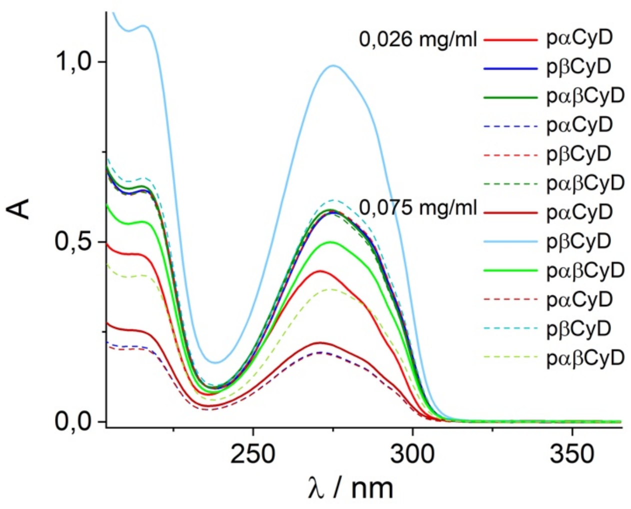

4) Calibration curves of the drugs BIC and CBX to determine the molar absorption coefficient

The graphs in Figure S3 show the absorption and CD spectra of drug solutions in 1 cm cuvet that were obtained preparing films from ethanolic solutions with known drug amount subsequently dissolved with aqueous carrier solutions containing **10 mg/ml polymer** kept stirring for 48 hrs. The spectra were corrected for the absorption or CD signal of the empty polymer. We plotted the absorbance at selected wavelengths as a function of the drug concentration to obtain the molar absorption coefficient upon linear fitting of the data. The blue BIC curves in a-c and the green curves in d-e refer to BIC and CBX in water, respectively.

Figure S3

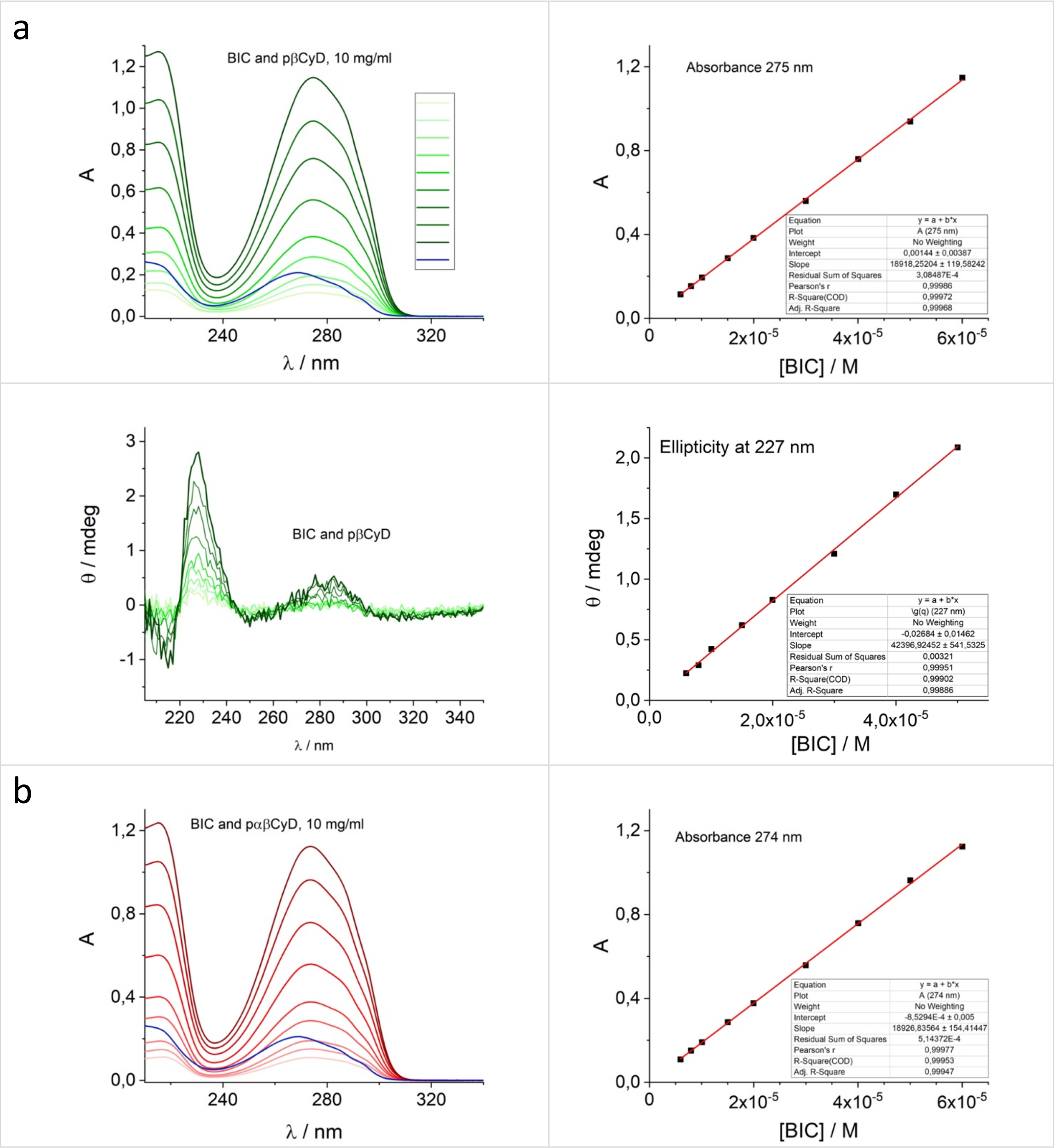

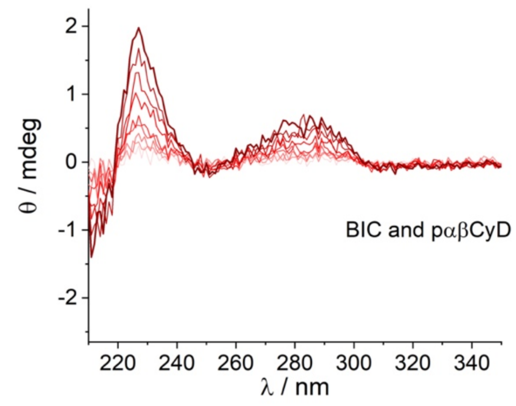

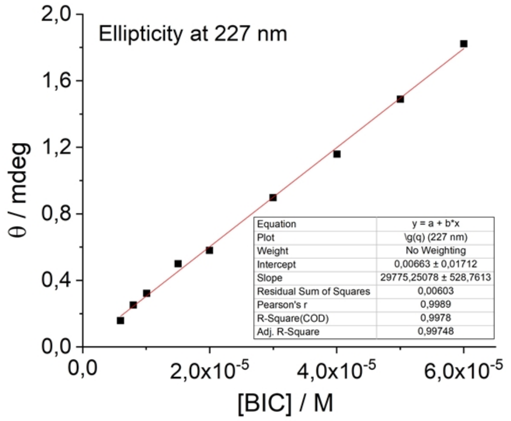

c

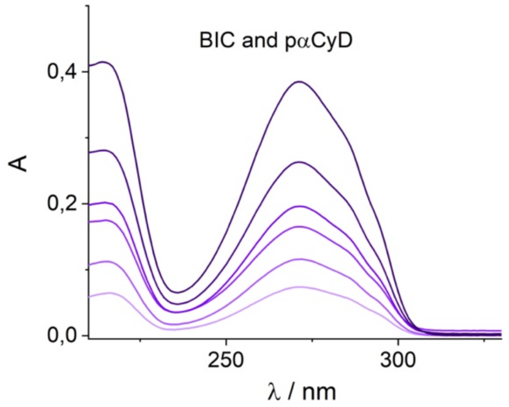

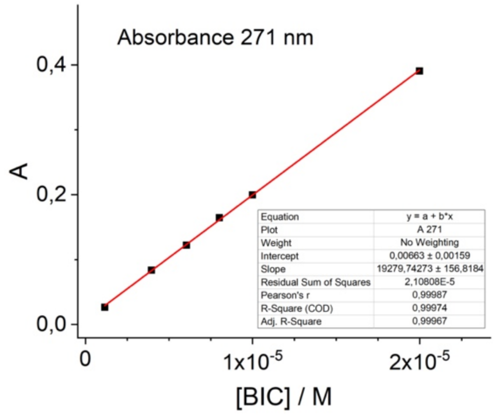

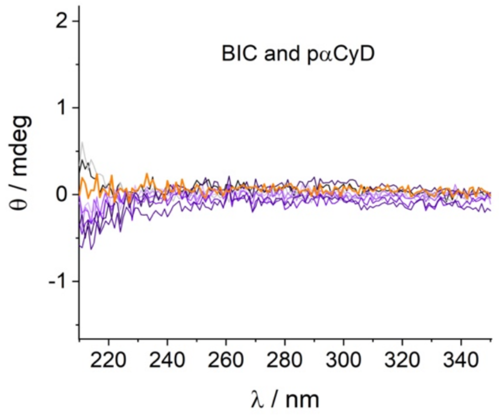

Not available

d

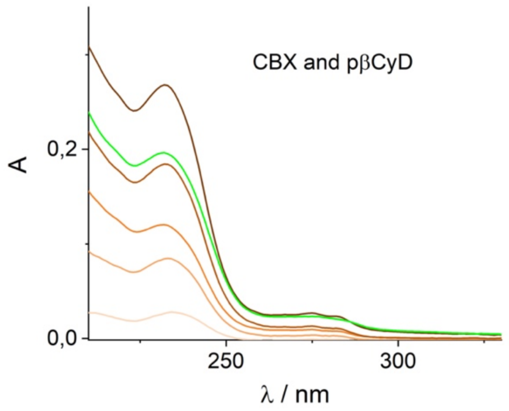

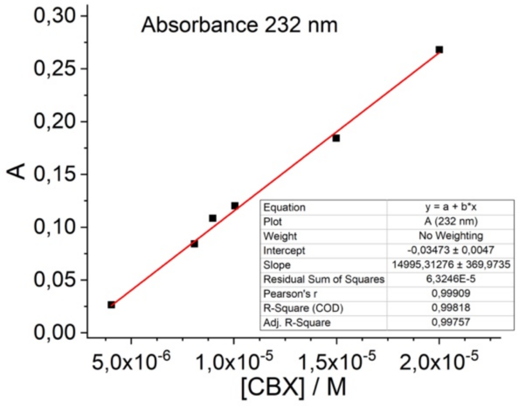

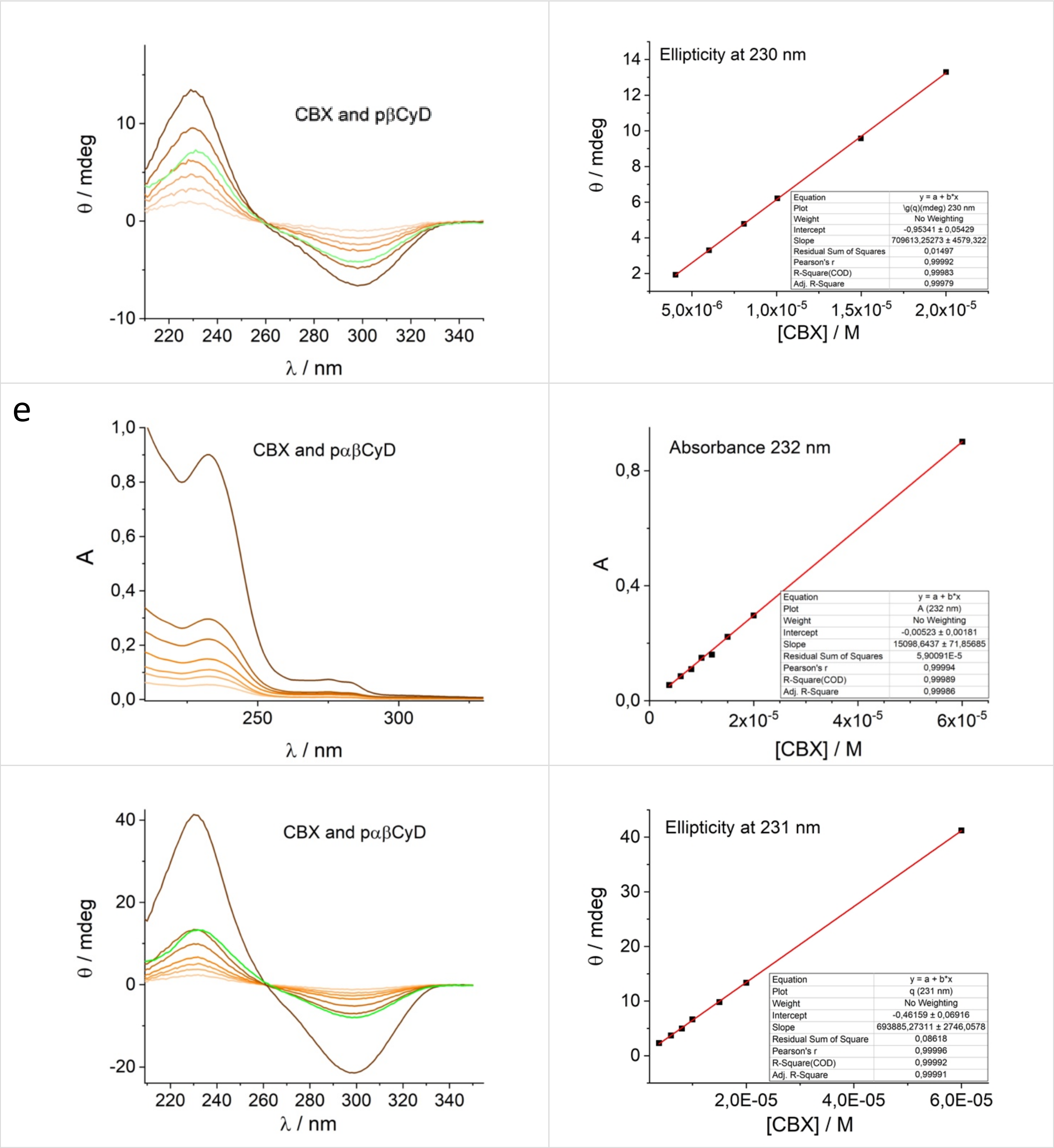

CBX and pβCyD
θ / mdeg
λ / nm
Ellipticity at 230 nm
θ / mdeg
[CBX] / M
Equation y = a + b*x
Plot \g(q)(mdeg) 230 nm
Weight No Weighting
Intercept -0,95341 ± 0,05429
Slope 709613,25273 ± 4579,322
Residual Sum of Squares 0,01497
Pearson's r 0,99992
R-Square(COD) 0,99983
Adj. R-Square 0,99979
e
CBX and pαβCyD
A
λ / nm
Absorbance 232 nm
A
[CBX] / M
Equation y = a + b*x
Plot A (232 nm)
Weight No Weighting
Intercept -0,00523 ± 0,00181
Slope 15098,6437 ± 71,85685
Residual Sum of Squares 5,90091E-5
Pearson's r 0,99994
R-Square(COD) 0,99989
Adj. R-Square 0,99986
CBX and pαβCyD
θ / mdeg
λ / nm
Ellipticity at 231 nm
θ / mdeg
[CBX] / M
Equation y = a + b*x
Plot q (231 nm)
Weight No Weighting
Intercept -0,46159 ± 0,06916
Slope 693885,27311 ± 2746,0578
Residual Sum of Square 0,08618
Pearson's r 0,99996
R-Square(COD) 0,99992
Adj. R-Square 0,99991

5) Titration Fluorescence spectra to determine the binding constants of BIC in pαCyD and pαβCyD

Figure S4

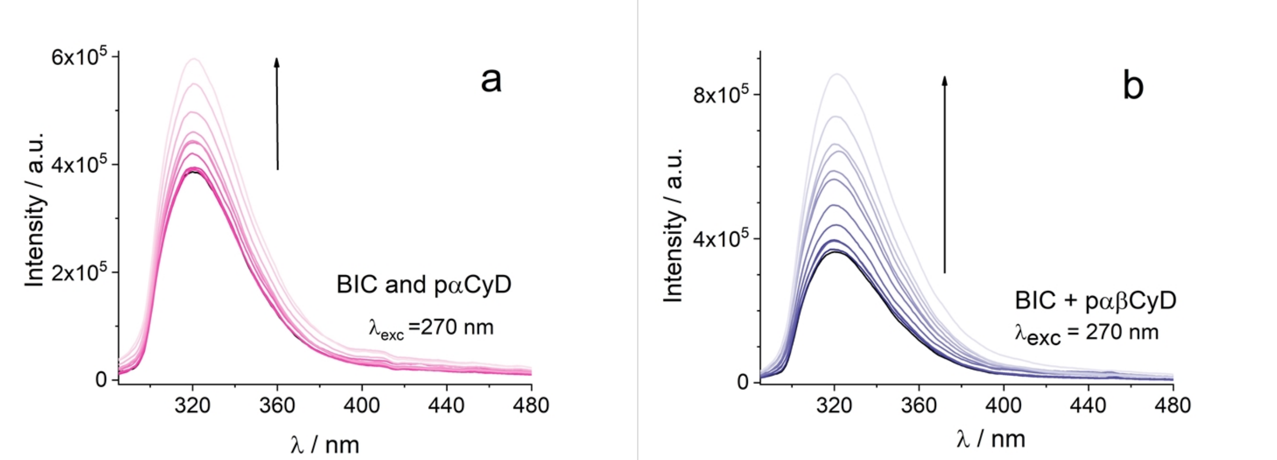

Titration Fluorescence spectra used to define the binding model and calculate the binding constants of BIC with the selected polymers; [BIC] = 4 μM; for the polymers: (a) 4-700 μM [αCyD]; (b) 6-350 μM [αβCyD].

Titration of BIC with the polymer pβCyD: (c) Comparison of the experimental and calculated fluorescence intensity values at selected wavelengths; (d) species distribution vs total βCyD concentration and (e) calculated spectra of free and complexed BIC.

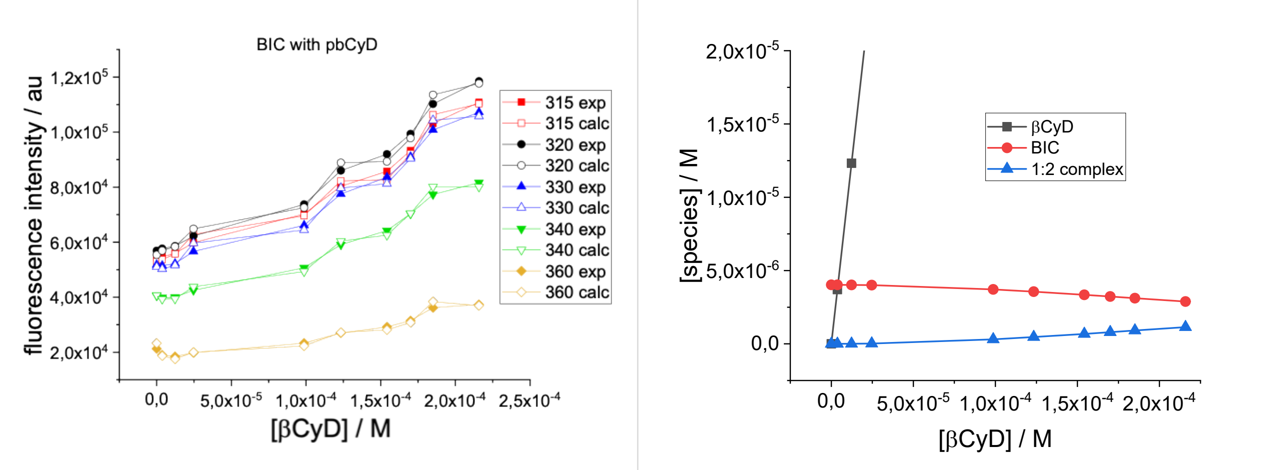

c

d

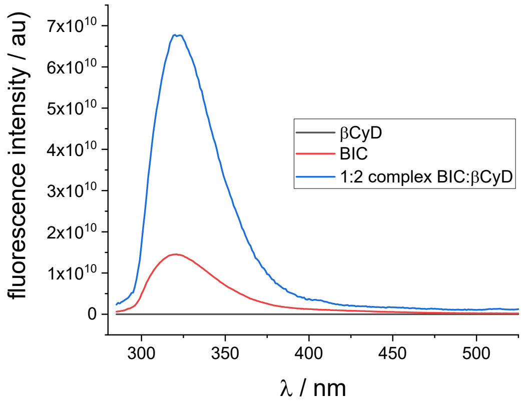

e

6) DFT calculations

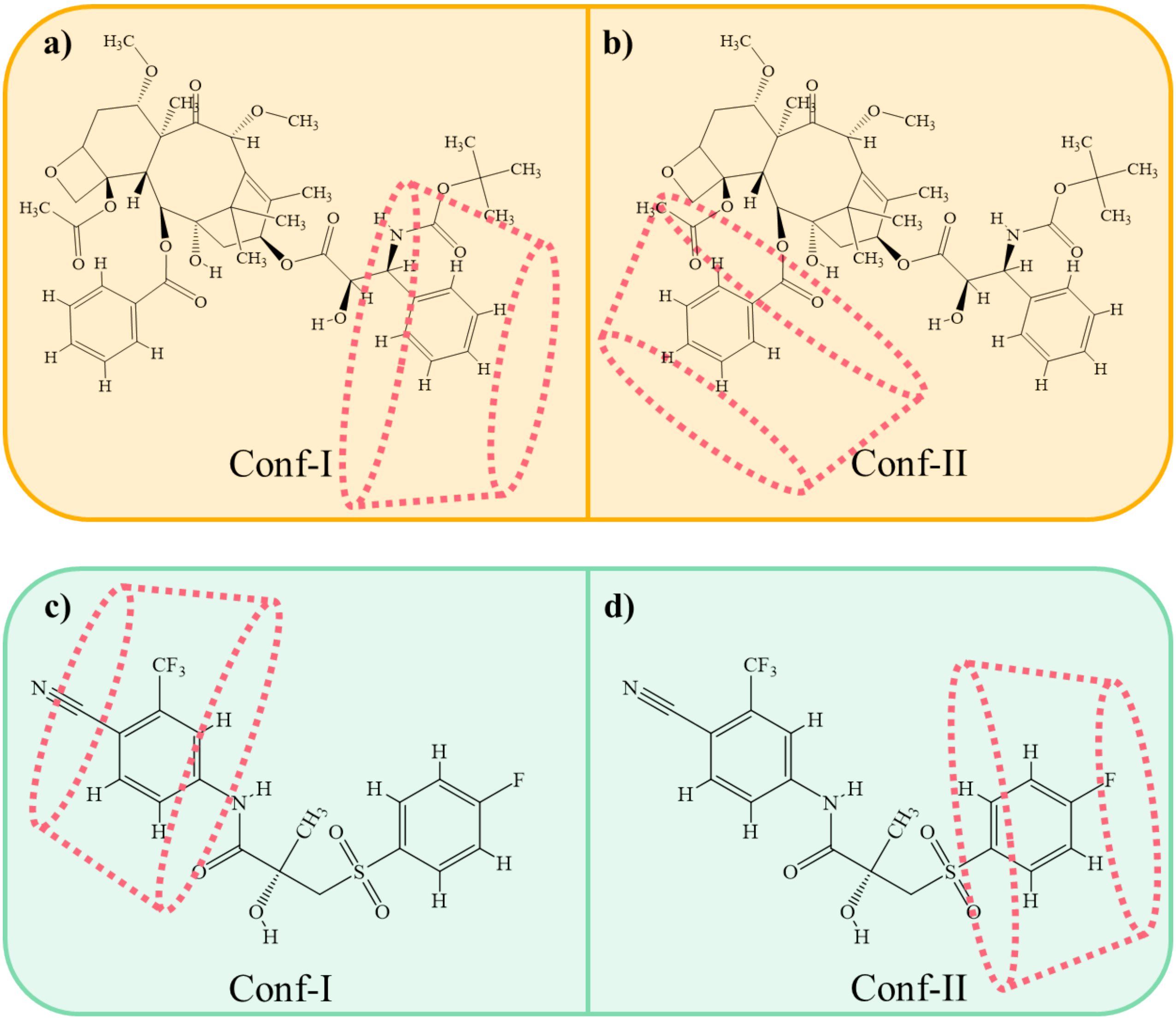

Figure S5 Schematics of the 1:1 molecular host-guest supramolecular complexes of β-CyD with a) and b) CBX (two structures investigated); c) and d) S-BIC (two structures investigated, same for R-BIC).

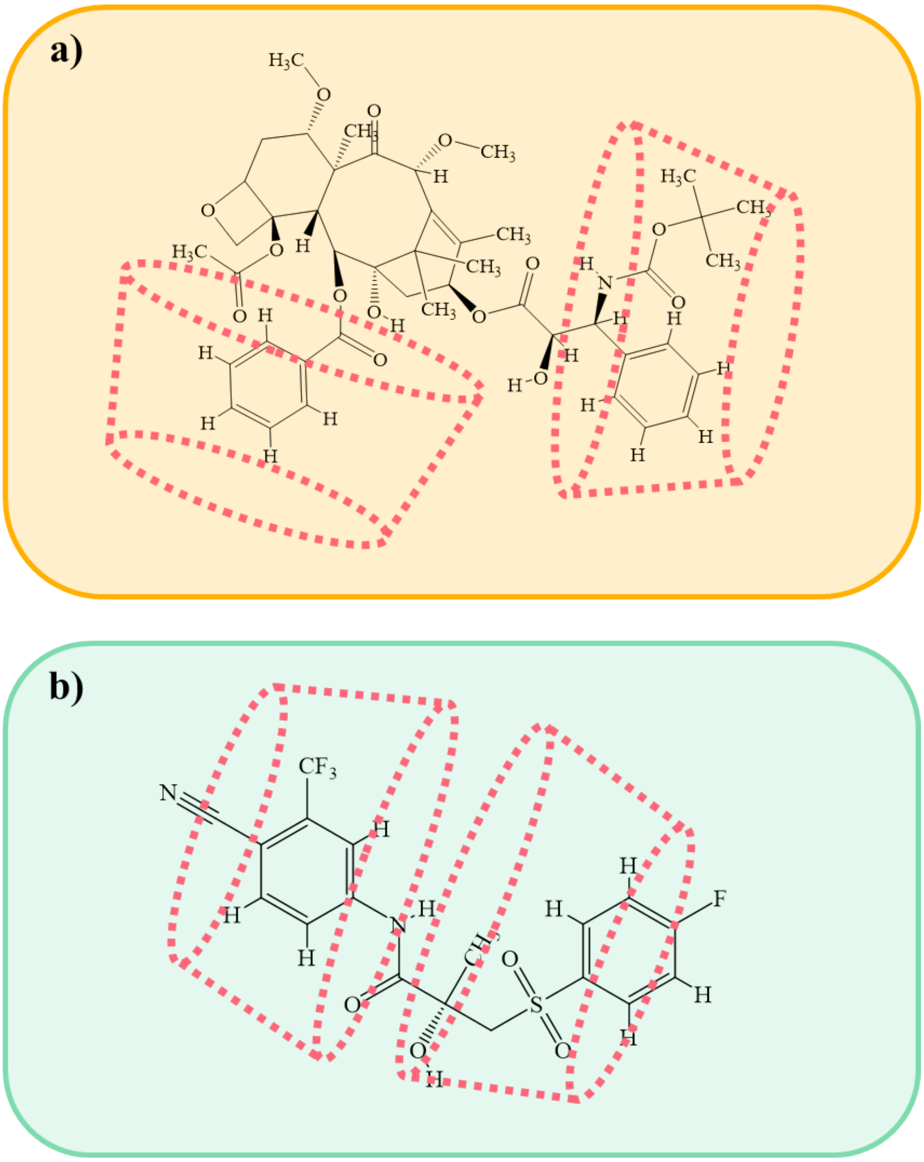

Figure S6 Schematics of the 1:2 molecular host-guest supramolecular complexes of two β-CyD with a) CBX; b) S-BIC (same for R-BIC).

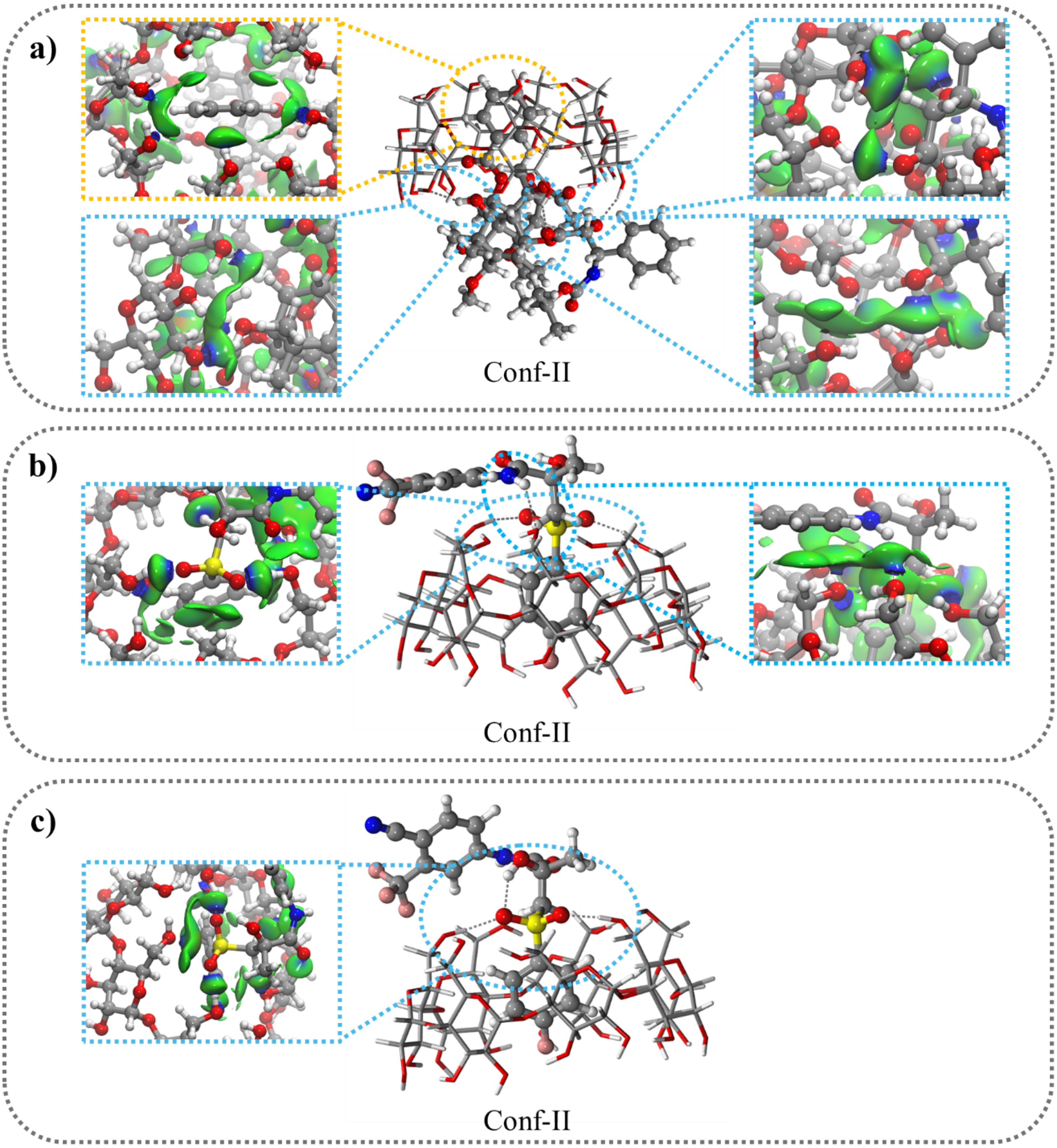

Figure S7 Optimized structures for 1:1 stoichiometry (Conf II) of βCyD and: a) CBX; b) S-BIC; c) R-BIC; with color-filled $\delta g^{inter}(\rho)$ projections (isovalue 0.0055 a.u.). vdW and HBs interactions are highlighted by yellow and blue circle respectively.

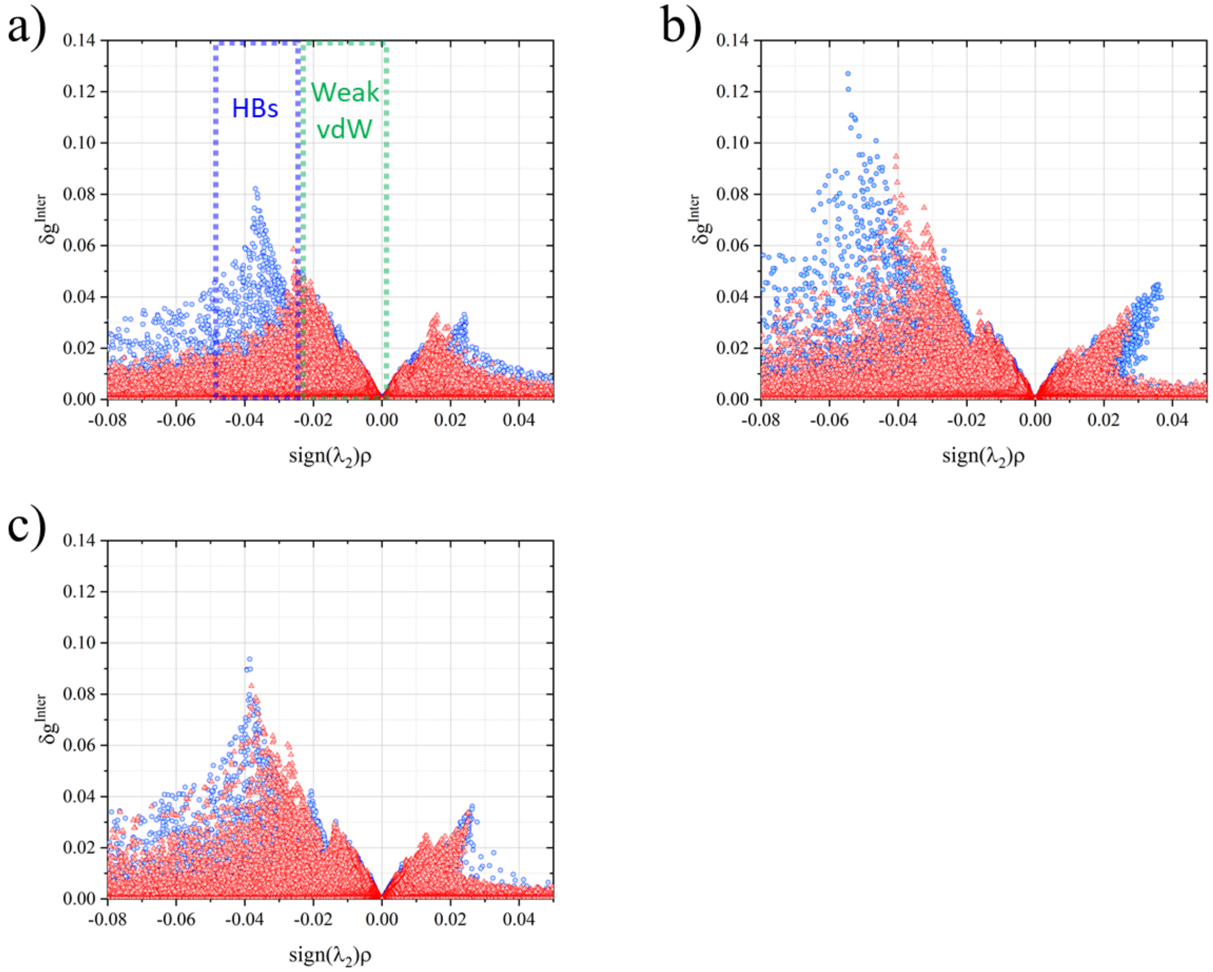

Figure S8 δg^{inter} plot for 1:1 stoichiometry of βCyD and: a) CBX (blue circle Conf-I; red triangle Conf-II); b) S-BIC (blue circle Conf-I; red triangle Conf-II); c) R-BIC (blue circle Conf-I; red triangle Conf-II).

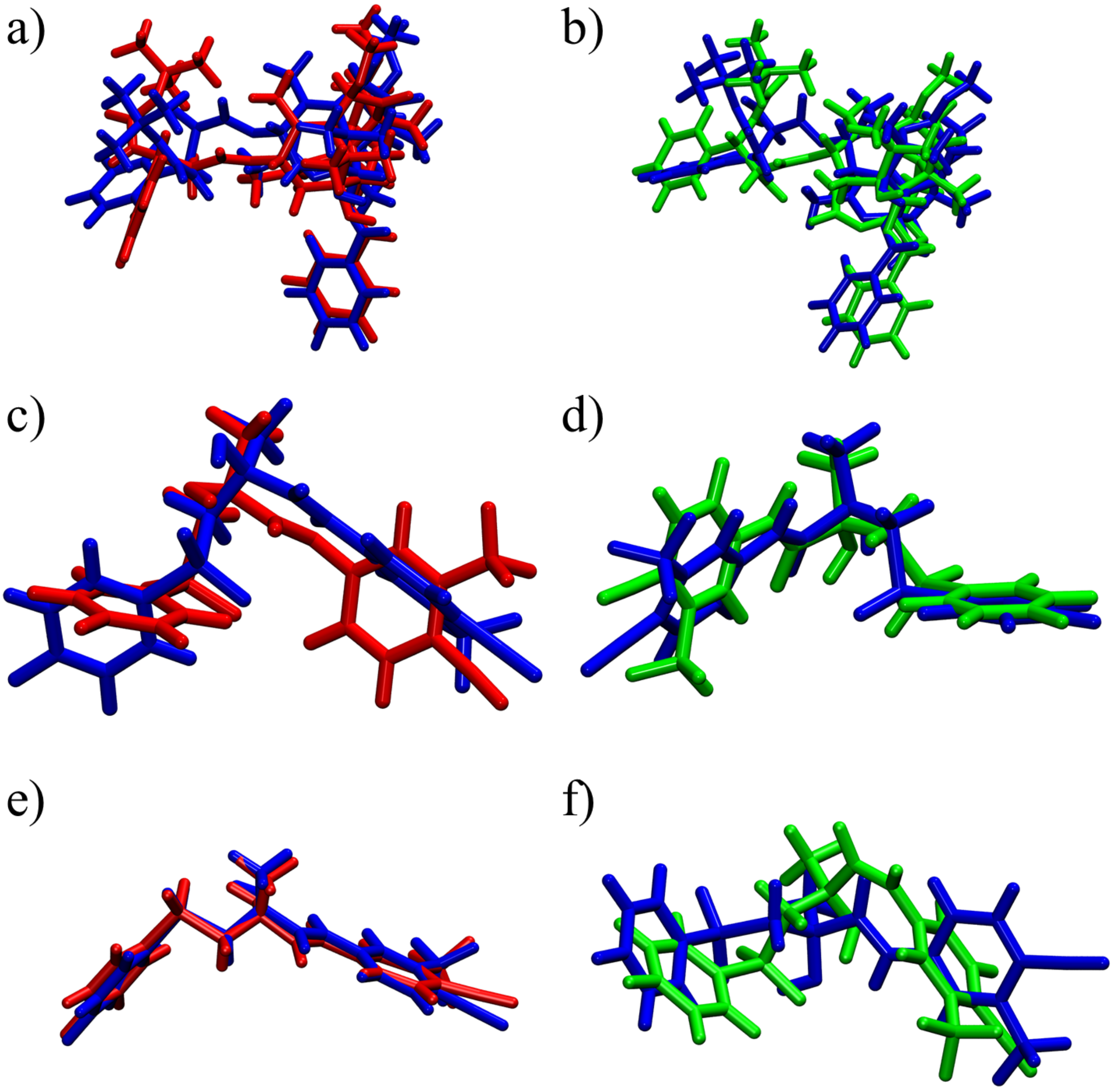

Figure S9 comparison between relax structure of guest molecules (blue) and guest molecules in Conf-I (red) and Conf-II complexes (green); a) and b) CBX; c) and d) S-BIC; e) and f) R-BIC.

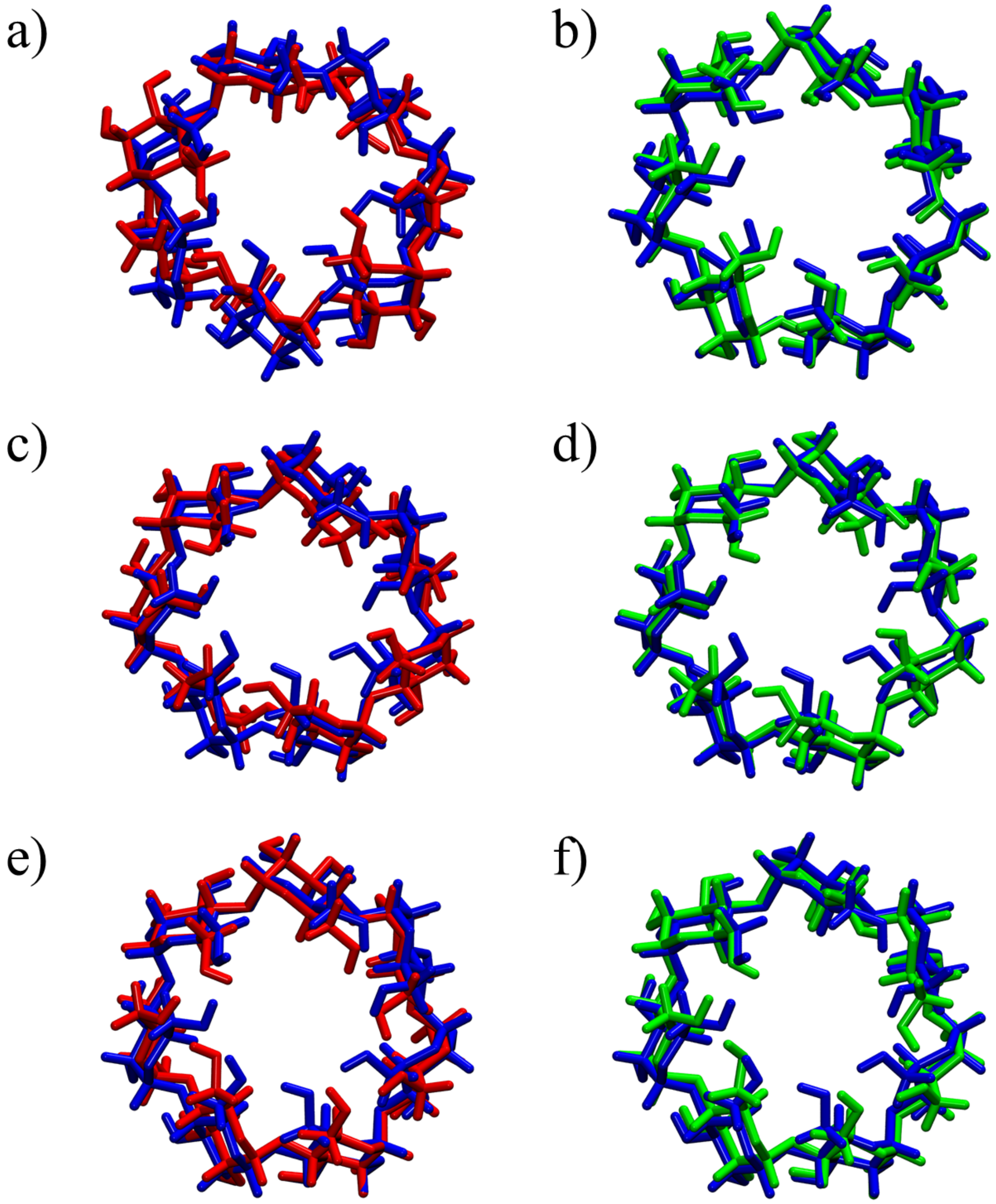

Figure S10 comparison between relax structure of βCyD molecule (blue) and βCyD molecules in Conf-I (red) and Conf-II complexes (green); a) and b) CBX@βCyD; c) and d) S-BIC@βCyD; e) and f) R-BIC@βCyD.

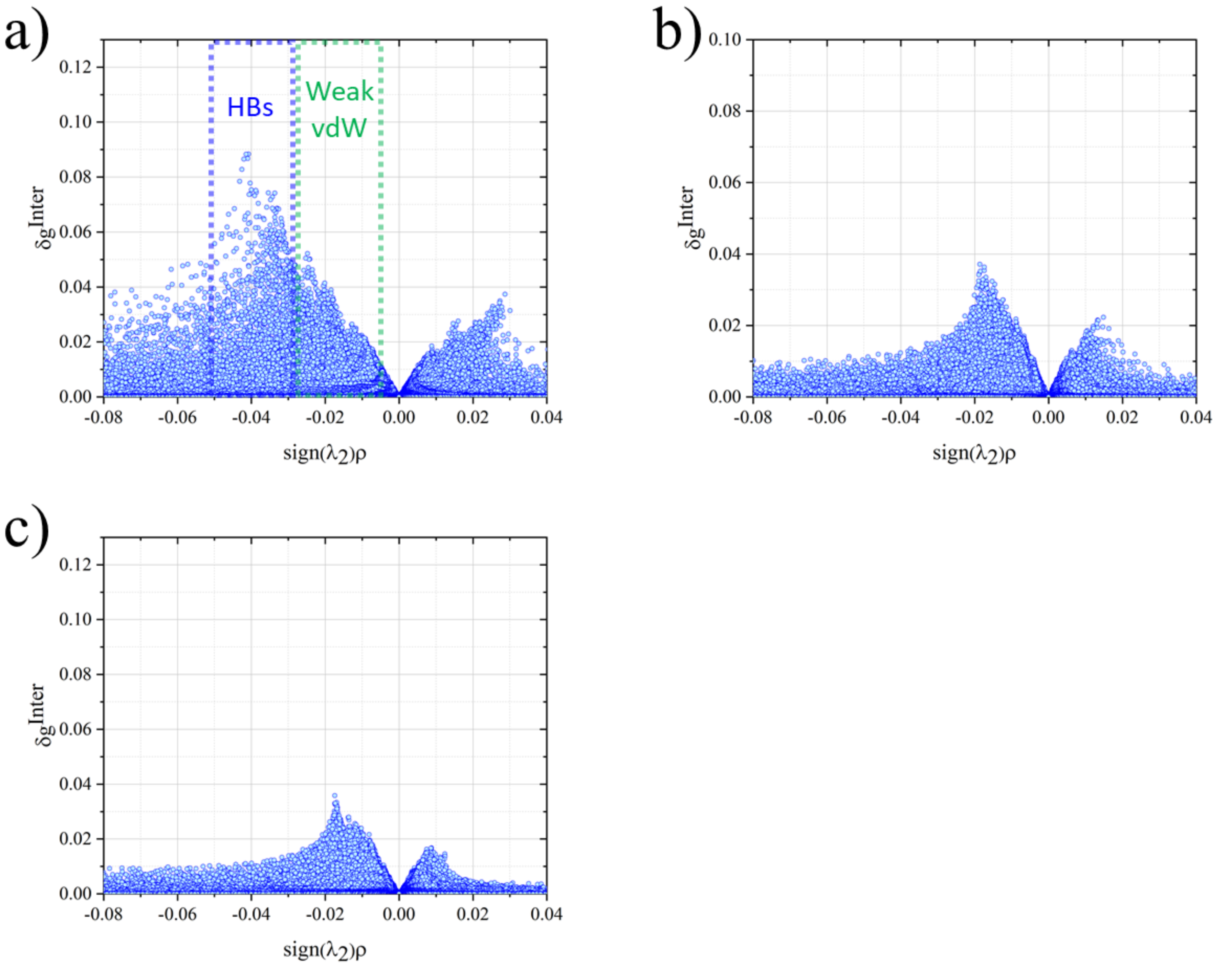

Figure S11 δg^{inter} plot for 1:2 stoichiometry of βCyD with: a) CBX; b) S-BIC; c) R-BIC.

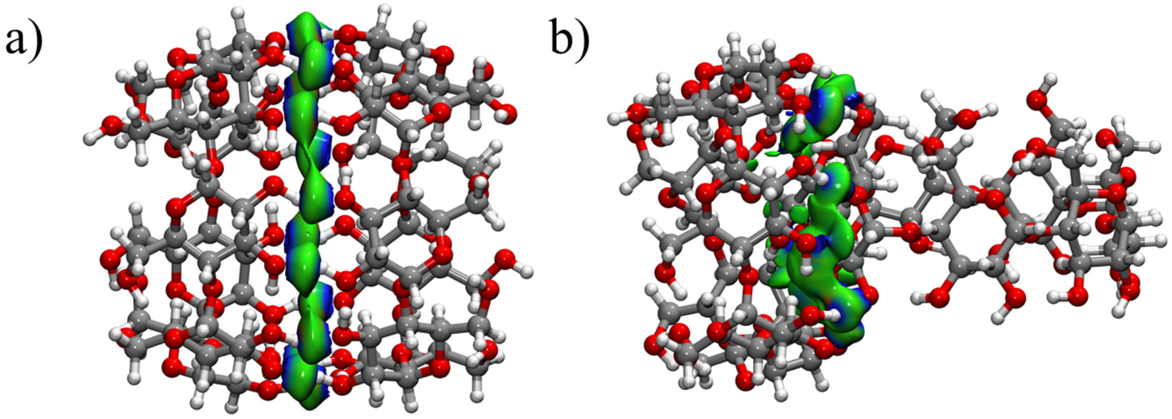

Figure S12 Optimization structures of cluster formed by 2 βCyD without out guest molecules with color-filled $\delta g^{inter}(\rho)$ plot (isovalue 0.0055 a.u.); structure a) is related to the relaxion of cluster formed with BIC; structure b) is related to the relaxion of cluster formed with CBX.

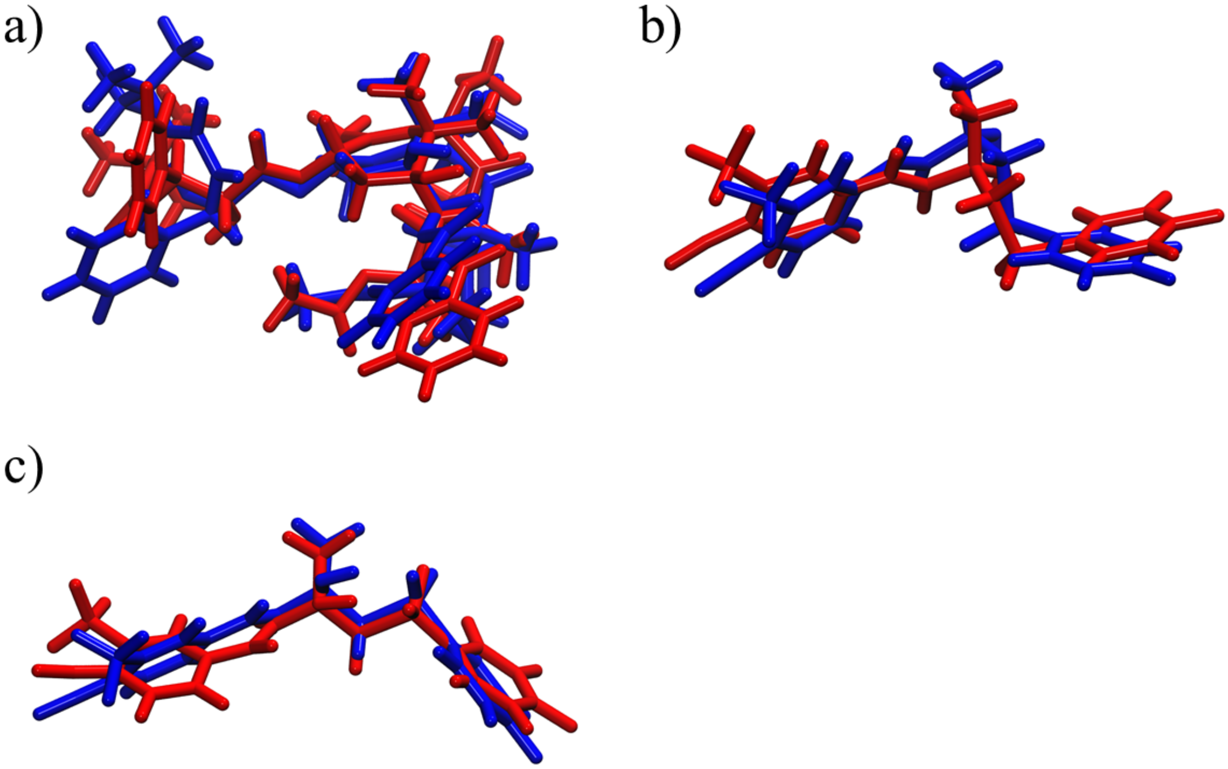

Figure S13 comparison between relax structure of guest molecules (blue) and guest molecules (red) in complexes; a) CBX; b) S-BIC; c) R-BIC.

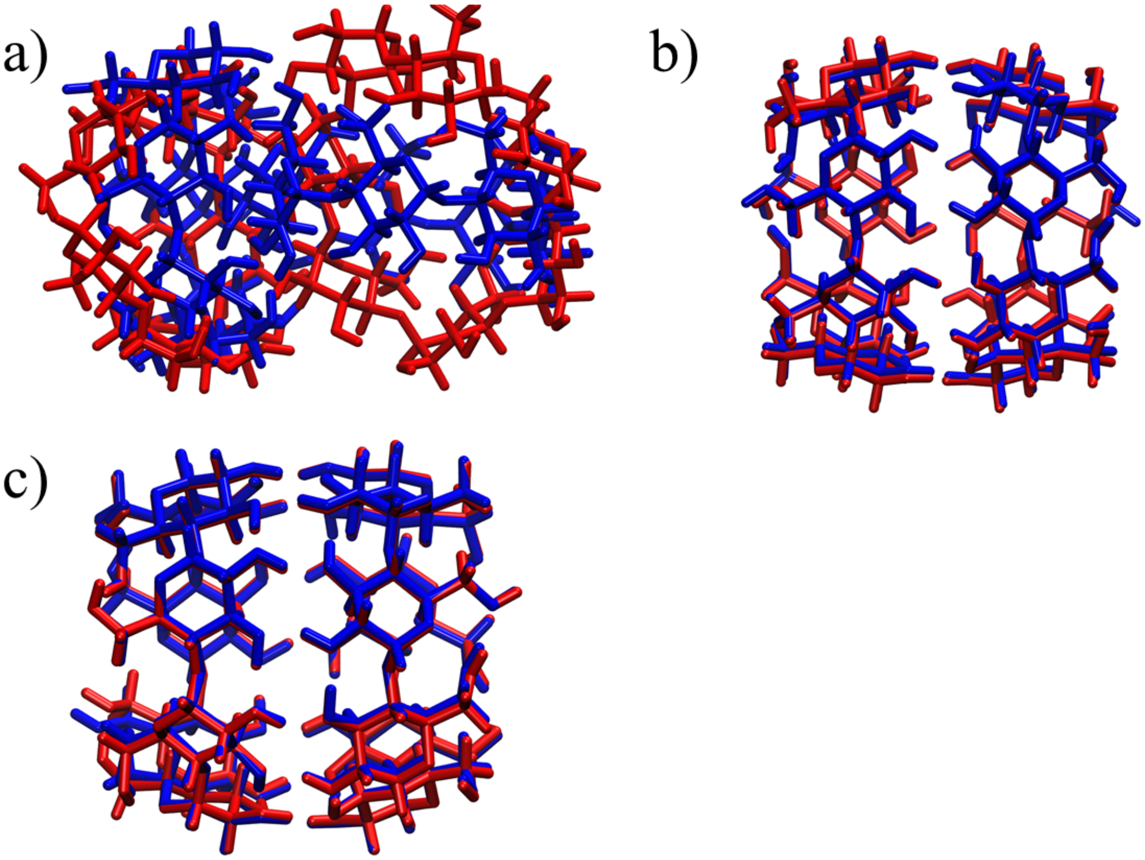

Figure S14 comparison between relax structure of βCyD molecule (blue) and βCyD molecules in complexes; a) CBX@βCyD; b) S-BIC@βCyD; c) R-BIC@βCyD.

Table S2. Absorption data: λ, *f* (oscillator strength) and *R* (optical rotatory strength) for the first 5 states.

	**R-BIC**			**S-BIC**			**CBX**		
**State**	**λ (nm)**	***f***	***R***	**λ (nm)**	***f***	***R***	**λ (nm)**	***f***	***R***
**1**	256.0	0.567	55.19	256.0	0.566	-55.40	288.7	0.00	-35.29
**2**	243.9	0.118	5.30	243.9	0.118	-5.37	240.7	0.03	-2.55
**3**	229.8	0.008	2.81	229.9	0.008	-2.62	226.5	0.01	-10.47
**4**	228.1	0.000	-0.45	228.1	0.000	0.45	226.5	0.00	0.23
**5**	206.1	0.173	-15.19	206.1	0.173	15.17	222.1	0.31	24.44
	**2βCyd-R-BIC**			**2βCyd-S-BIC**			**2βCyd-CBX**		
**1**	260.2	0.507	-35.53	258.2	0.455	5.74	291.7	0.00	-41.76
**2**	250.0	0.066	0.53	246.6	0.087	-0.78	244.0	0.26	-0.30
**3**	234.7	0.006	2.36	230.9	0.001	97.62	231.4	0.01	0.35
**4**	231.5	0.007	-14.87	223.6	0.006	-19.28	230.9	0.00	-7.66
**5**	210.6	0.171	51.18	209.8	0.114	18.38	225.7	0.27	-6.69

7) Titration data analysis of Ce6 with pbCyD

Figure S15: Output for the binding analysis of the Absorption and Fluorescence spectra and global analysis of the fluorescence decay of Ce6 with different polymer amounts

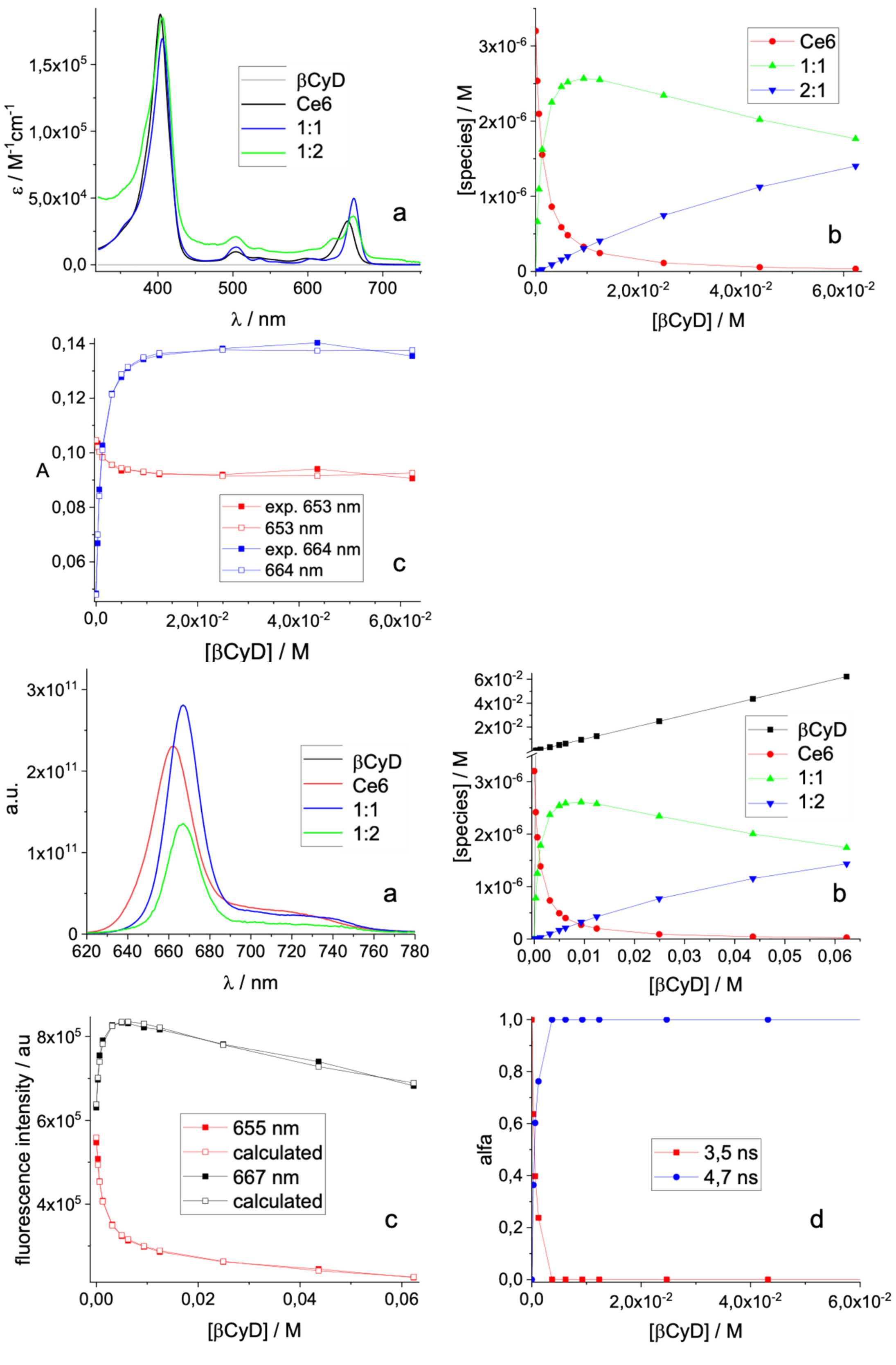

8) co-encapsulation of BIC and CBX

Figure S16: stability test of the mixtures

Absorption spectra of BIC with pβCyD (blue), pαβCyD (red) and pαβCyD (green) measured at time 0 (lines) and after 28 days (dashed).

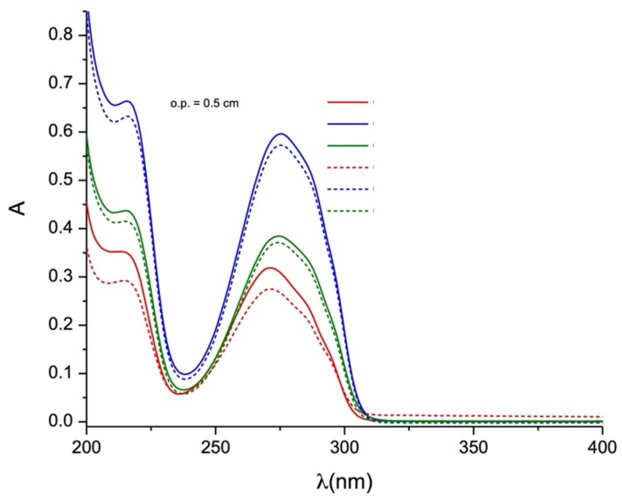

Absorption spectra of CBX with pβCyD (blue), pαβCyD (red) and pαβCyD (green) measured at time 0 (lines) and after 28 days (dashed).

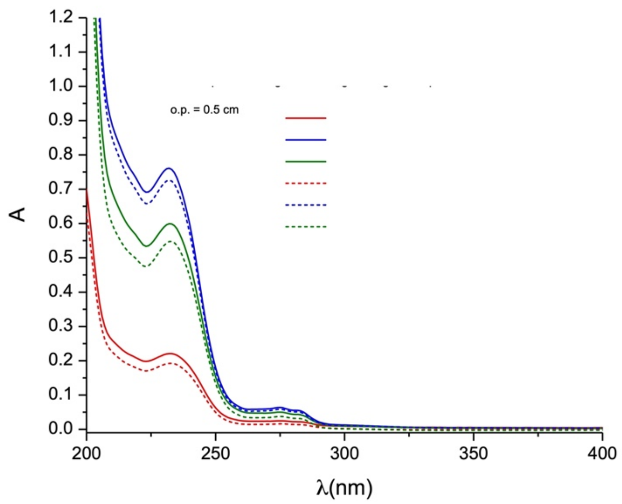

Absorption spectra of CBX and BIC with pβCyD (blue), pαβCyD (red) and pαβCyD (green) measured at time 0 (lines) and after 28 days (dashed).

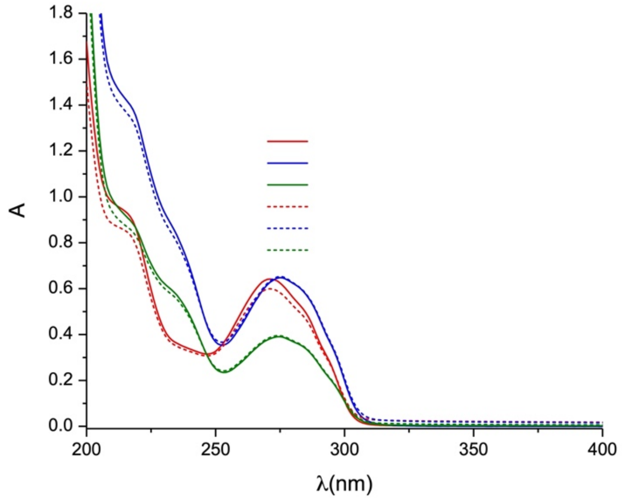

Figure S17: results for the global analysis of the fluorescence decay of Ce6 in the presence of different amounts of polymer and CBX

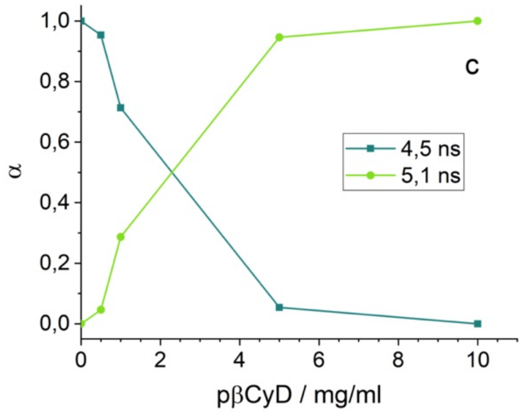

Supplement: RA-013-D3RA01782F-s001 [file RA-013-D3RA01782F-s001.pdf]
